# Elucidation of an mTORC2-PKC-NRF2 pathway that sustains the ATF4 stress response and identification of Sirt5 as a key ATF4 effector

**DOI:** 10.1038/s41420-022-01156-5

**Published:** 2022-08-13

**Authors:** Ruizhi Li, Kristin F. Wilson, Richard A. Cerione

**Affiliations:** 1 Department of Molecular Medicine, Cornell University, Ithaca, NY 14853 USA; 2 Department of Chemistry and Chemical Biology, Cornell University, Ithaca, NY 14853 USA

**Keywords:** Stress signalling, Breast cancer

## Abstract

Proliferating cancer cells are dependent on glutamine metabolism for survival when challenged with oxidative stresses caused by reactive oxygen species, hypoxia, nutrient deprivation and matrix detachment. ATF4, a key stress responsive transcription factor, is essential for cancer cells to sustain glutamine metabolism when challenged with these various types of stress. While it is well documented how the *ATF4* transcript is translated into protein as a stress response, an important question concerns how the *ATF4* message levels are sustained to enable cancer cells to survive the challenges of nutrient deprivation and damaging reactive oxygen species. Here, we now identify the pathway in triple negative breast cancer cells that provides a sustained ATF4 response and enables their survival when encountering these challenges. This signaling pathway starts with mTORC2, which upon sensing cellular stresses arising from glutamine deprivation or an acute inhibition of glutamine metabolism, initiates a cascade of events that triggers an increase in *ATF4* transcription. Surprisingly, this signaling pathway is not dependent on AKT activation, but rather requires the mTORC2 target, PKC, which activates the transcription factor Nrf2 that then induces *ATF4* expression. Additionally, we identify a sirtuin family member, the NAD^+^-dependent de-succinylase Sirt5, as a key transcriptional target for ATF4 that promotes cancer cell survival during metabolic stress. Sirt5 plays fundamental roles in supporting cancer cell metabolism by regulating various enzymatic activities and by protecting an enzyme essential for glutaminolysis, glutaminase C (GAC), from degradation. We demonstrate that ectopic expression of Sirt5 compensates for knockdowns of *ATF4* in cells exposed to glutamine deprivation-induced stress. These findings provide important new insights into the signaling cues that lead to sustained ATF4 expression as a general stress-induced regulator of glutamine metabolism, as well as highlight Sirt5 an essential effector of the ATF4 response to metabolic stress.

## Introduction

Tumorigenesis requires adequate ATP production, stable redox homeostasis, rapid biosynthesis and adaptation to the complex tumor environment. Metabolic reprogramming plays an important role by ensuring redox homeostasis, adaptation to various types of stresses, and maintaining the balance between catabolism and anabolism [1]. Most cancer cells increase glucose uptake and divert glucose from OXPHOS to lactate secretion, regardless of oxygen availability. Since carbons from glycolytic flux are shunted into *de novo* synthesis pathways or secreted as lactate, and because TCA cycle generated acetyl-CoA is essential for lipogenesis, cancer cells depend upon an exogenous supply of glutamine to produce TCA cycle intermediates (i.e., the Warburg effect) [2]. Glutaminase (GLS) catalyzes the first step in glutamine metabolism, the hydrolysis of glutamine to glutamate with the production of ammonia. The C-terminal splice variant of GLS, glutaminase C (GAC), is often highly expressed in breast cancer cells and helps satisfy their glutamine addiction [3].

Sirtuin5 (Sirt5) is an NAD^+^-dependent lysine deacylase that catalyzes lysine succinylation, malonylation and glutarylation [4, 5]. It supports glutamine metabolism and oncogenesis in breast cancer [6] by catalyzing lysine-desuccinylation on GAC to protect it from ubiquitin-mediated degradation. Although Sirt5 levels are increased in various types of cancer, how its expression is mediated in response to metabolic stress is not known.

The mechanistic Target of Rapamycin (mTOR) integrates growth factor signaling and nutrient levels with growth and survival and plays an essential role in the proliferation and metabolism of cancer cells. mTOR is found within two distinct complexes, mTORC1 and mTORC2. Numerous studies have shown that mTORC1 enhances protein translation and metabolism in response to growth factors and nutrients. While the function of mTORC2 is less well understood [7–10], it is known to act as a central regulator for the AGC family of kinases (AKT/PKC/SGK1), and to enhance metabolism through AKT by promoting glycolytic flux and amino acid transporter expression. mTORC2 also phosphorylates several PKC isoforms (α, β, γ, δ, ε, η, θ, and µ) [11]. PKC has been found to increase the abundance of Nuclear Factor Erythroid-derived 2-like 2 (Nrf2) [ref. 12–18], the key transcriptional factor to activate the antioxidant defense system; however, a role for mTORC2 in regulating redox balance and cellular metabolism via PKC has not been previously demonstrated.

Here, we report that mTORC2 transmits signals which result in the upregulation of Activating Transcription Factor 4 (ATF4) transcription via the PKC-Nrf2 axis upon oxidative stress. ATF4 is a key stress-induced transcription factor whose translation is markedly increased during the integrated stress response (ISR) [19]. Growing evidence suggests that a pro-survival role of ATF4 is to regulate gene-encoding proteins important for the elevation of glutamine metabolism in cancer [20–26], although in certain contexts ATF4 can also trigger cell death [27]. We now describe how mTORC2-mediated *ATF4* transcription is necessary to replenish *ATF4* levels to maintain ATF4 function and enable cancer cells to survive sustained metabolic stress. Additionally, we show that *Sirt5* is a transcriptional target of ATF4 necessary for its pro-survival role in response to oxidative stress and conditions that negatively impact glutamine metabolism in cancer cells. Sirt5 expression ensures the survival of breast cancer cells and restores their viability upon the knock-down of *ATF4*. Together, these results highlight a pro-survival mTORC2-dependent signaling pathway that culminates in the sustained expression of ATF4 and the upregulation of its transcriptional target *Sirt5* to promote adaptive compensation to metabolic and oxidative challenges.

## Results

### Oxidative stress causes an upregulation of ATF4 transcription

**Fig. 1:**
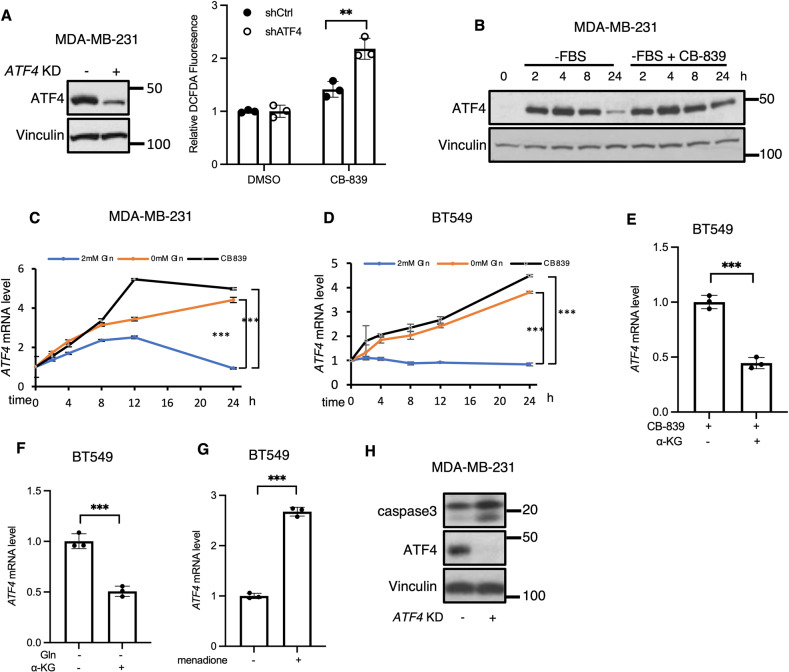
*ATF4* gene expression is the target of oxidative stress signaling in breast cancer. **A** ROS detection assay in control or *ATF4* KD MDA-MB-231 cells ± CB-839 (5 μM) for 24 h. Data shown are representative of two independent experiments and expressed as means ± SD for triplicate measurements. **B** Western blot analysis of whole cell lysates (WCL) from MDA-MB-231 cells ± CB-839 (1 μM) at indicated times. Blots are representative of two independent experiments. **C**, **D** RT-qPCR quantification of *ATF4* mRNA in MDA-MB-231 and BT549 cells ± CB-839 (1 μM) or ± glutamine (2 mM) at the indicated times. Data shown are representative of two independent experiments and expressed as means ± SD for triplicate measurements. **E**, **F** RT-qPCR quantification of *ATF4* mRNA in BT549 cells treated with CB-839 (1 μM) or without glutamine ± DM-αKG (2 mM) for 24 h. Data shown are representative of two independent experiments and expressed as means ± SD for triplicate measurements. **G** RT-qPCR quantification of *ATF4* mRNA in BT549 cells treated with or without menadione (2 μM) for 24 h. Data shown are representative of three independent experiments and expressed as means ± SD for triplicate measurements. **H** Western blot analysis of WCL from MDA-MB-231 cells cultured in glutamine free condition for 48 h. Blots are representative of two independent experiments.

The stress-inducible transcription factor, ATF4, has been suggested to play an essential role in the ability of cancer cells to adapt to multiple challenges, including metabolic stress [22, 28–32]. ATF4 is known to be rapidly translated as part of the ISR Pathway through the regulatory effects of eIF2α [19]. Therefore, we sought to understand what role ATF4 plays in the ability of cancer cells to adapt to the metabolic requirements of increased glutamine metabolism accompanying the “Warburg effect” by using a GAC inhibitor, CB-839, to disrupt glutaminolysis. We first tested how ATF4 affected ROS levels when MDA-MB-231 cells were challenged with CB-839. *ATF4* knockdown cells or control cells were incubated with CB-839 for 24 h in the presence of serum-free media. CB-839 treatment increased cellular ROS, as measured by DCFDA fluorescence, with the knock-down of *ATF4* expression further enhancing this effect (Fig. 1A). Additionally, supplementation with antioxidants promoted cell viability in *ATF4* knockdown cells (Fig. S1A).

Nutrient deprivation is sufficient to induce the rapid translation of *ATF4*. Therefore, we examined whether CB-839 provided any additional effects on ATF4 expression when combined with serum starvation. MDA-MB-231 cells were incubated with either serum-free media, or serum-free media plus CB-839 for up to 24 h and then probed for ATF4 protein expression. Each condition induced ATF4 expression significantly by 1 h of treatment, with peak levels occurring after 4–8 h (Fig. 1B). However, CB-839 treatment had a prolonged effect on ATF4 expression through 24 h, compared to serum deprivation alone. We repeated the time course experiments probing for *ATF4* message levels by qPCR. Strikingly, while ATF4 transcript levels remained low in both serum-starved MDA-MB-231 cells (Fig. 1C) and BT549 cells (Fig. 1D), *ATF4* message levels rose significantly in cells treated with CB-839 or when undergoing glutamine withdrawal. The co-treatment of cells with Actinomycin D (ActD, an RNA polymerase inhibitor) and either CB-839 or glutamine withdrawal for 24 h eliminated any accumulation of *ATF4* transcript or protein (Fig. S1B–D), suggesting that ATF4 expression was promoted via a transcriptional mechanism under conditions of metabolic stress, contrasting with mechanisms for the increase in ATF4 expression that occur during nutrient deprivation.

To further confirm the specificity of this transcriptional response of *ATF4*, we examined whether the metabolic stress imposed by CB-839 and glutamine withdrawal might be reduced by supplying cells with a cell permeable analog of α-keto-glutarate (αKG), the product of glutaminolysis that is generated downstream of the GAC-catalyzed production of glutamate. Indeed, the co-treatment of BT549 cells or MBA-MB-231 cells with dimethyl-αKG (DM-αKG) under conditions that negatively impacted glutaminolysis for 24 h significantly lowered the stress-induced increases in *ATF4* transcript (Figs. 1E, F; Fig. S1E). We further examined whether this ATF4 response occurred when cells were treated with menadione to generate cellular ROS and again found that *ATF4* transcript levels were increased (Fig. 1G). To then ascertain the functional impact of *ATF4* gene expression on cell survival, we grew MBA-MB-231 cells in glutamine-free media, with or without the knock-down of *ATF4*, then collected samples after 48 h. When probing for activated caspase-3, we detected that *ATF4* knockdown cells showed activation of caspase-3 (p19 and p17) in glutamine-depleted media (Fig. 1H), suggesting the necessity of ATF4 for sustained survival. Together, these data demonstrate the presence of a metabolic stress-sensing pathway that helps to extend the lifetime of ATF4 in order to ensure the survival of breast cancer cells.

### mTORC2 signaling increases ATF4 expression in an AKT-independent manner

**Fig. 2:**
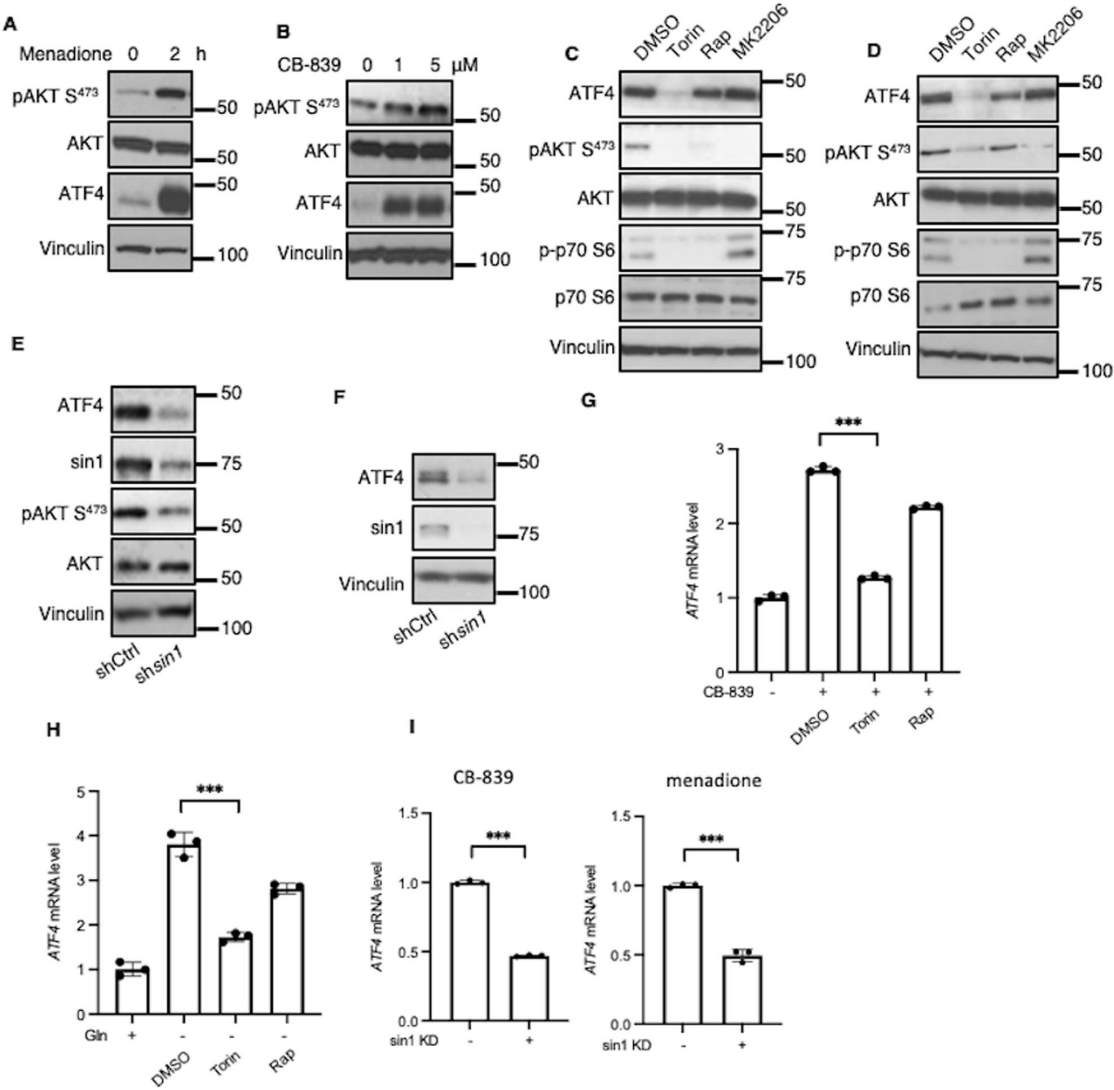
mTORC2 increases *ATF4* transcription independently of AKT. **A** Western blot analysis of WCL from MDA-MB-231 cells treated with menadione (50 μM) for 2 h. Blots are representative of two independent experiments. **B** Western blot analysis of WCL from MDA-MB-231 cells treated with CB-839 (1 μM) for 24 h. Blots are representative of three independent experiments. **C**, **D** Western blot analysis of WCL from MDA-MB-231 cells treated with CB-839 (1 μM) (**C**) or without glutamine (**D**)  ± Torin1 (500 nM), Rapamycin (50 nM) or MK2206 (5 μM) for 24 h. Blots are representative of three independent experiments. **E**, **F** Western blot analysis of WCL from control or *sin1* KD MDA-MB-231 cells cultured in glutamine-free medium (**E**) or treated with CB-839 (1 μM) (**F**) for 24 h. Blots are representative of two independent experiments. **G**, **H** RT-qPCR quantification of *ATF4* mRNA in MDA-MB-231 cells treated with CB-839 (1 μM) (**G**) or cultured ± glutamine (**H**) for 24 h with Torin1 (500 nM), Rapamycin (50 nM) or MK2206 (5 μM). Data shown are representative of three independent experiments and expressed as means ± SD for triplicate measurements. **I** RT-qPCR quantification of *ATF4* mRNA in control or *sin1* KD MDA-MB-231 cells treated with CB-839 (1 μM) or menadione (10 μM) for 24 h. Data shown are representative of two independent experiments and expressed as means ± SD for triplicate measurements.

We next set out to identify the signaling mechanisms responsible for increases in *ATF4* transcription that cancer cells need to survive metabolic stress. Because AKT has been shown to be activated via mTORC2 in response to oxidative stress [33], we used a phospho-specific AKT antibody to detect AKT phosphorylation upon menadione treatment of MDA-MB-231 cells. We found an increase in AKT phosphorylation at Ser^473^, the mTORC2-dependent phosphorylation site, that was accompanied by an increase in ATF4 (Fig. 2A). Treating cells with CB-839 also induced increases in AKT phosphorylation together with ATF4 expression (Fig. 2B).

We then tested the ability of the pan-mTOR inhibitor, Torin1, or the AKT inhibitor, MK2206, to affect ATF4 protein expression, in BT549 and MDA-MB-468 cells, and used the mTORC1 specific inhibitor, rapamycin, as a control. Menadione-induced ATF4 expression was largely blocked by Torin1, whereas rapamycin did not show a significant effect (Fig. S2A). Unexpectedly, MK2206 had little effect on the expression of ATF4. Cells were then treated with CB-839, either in the absence or presence of the mTOR or AKT inhibitors. Again, treatment with rapamycin or MK2206 had little on ATF4 levels, whereas Torin1 largely blocked its expression (Figs. 2C, S2B). The same was true under conditions of glutamine deprivation. ATF4 expression was blocked by Torin1, but only modestly decreased by rapamycin, and showed no change with MK2206 treatment (Fig. 2D). These results indicated that ATF4 expression is stimulated downstream of mTORC2 in an AKT-independent manner, thus uncoupling the metabolic stress-induced activation of AKT from the increased expression of ATF4. To confirm that the upregulation of ATF4 was mediated by mTORC2, we used short hairpin RNA to knock down *sin1*, a key component of mTORC2, and tested the effects on ATF4 expression in MDA-MB-231 and MDA-MB-468 cells. Knock-down of *sin1* decreased both AKT-mediated Ser^473^ phosphorylation and ATF4 expression under conditions of glutamine deprivation (Fig. 2E, S2C) and CB-839 treatment (Figs. 2F, S2C). Finally, we examined whether mTORC2 increased *ATF4* at a transcriptional level in response to perturbations in glutamine metabolism. Indeed, Torin1 suppressed *ATF4* transcript levels whereas rapamycin had no effect in MDA-MB-231 or BT549 cells treated with CB-839 or under glutamine-free conditions (Fig. 2G, H and S2D). Similarly, knocking down *sin1* decreased *ATF4* transcripts in MDA-MB-231 cells that were treated with CB-839, menadione or deprived of glutamine (Fig. 2I, S2E).

### mTORC2 promotes ATF4 expression via PKC

**Fig. 3:**
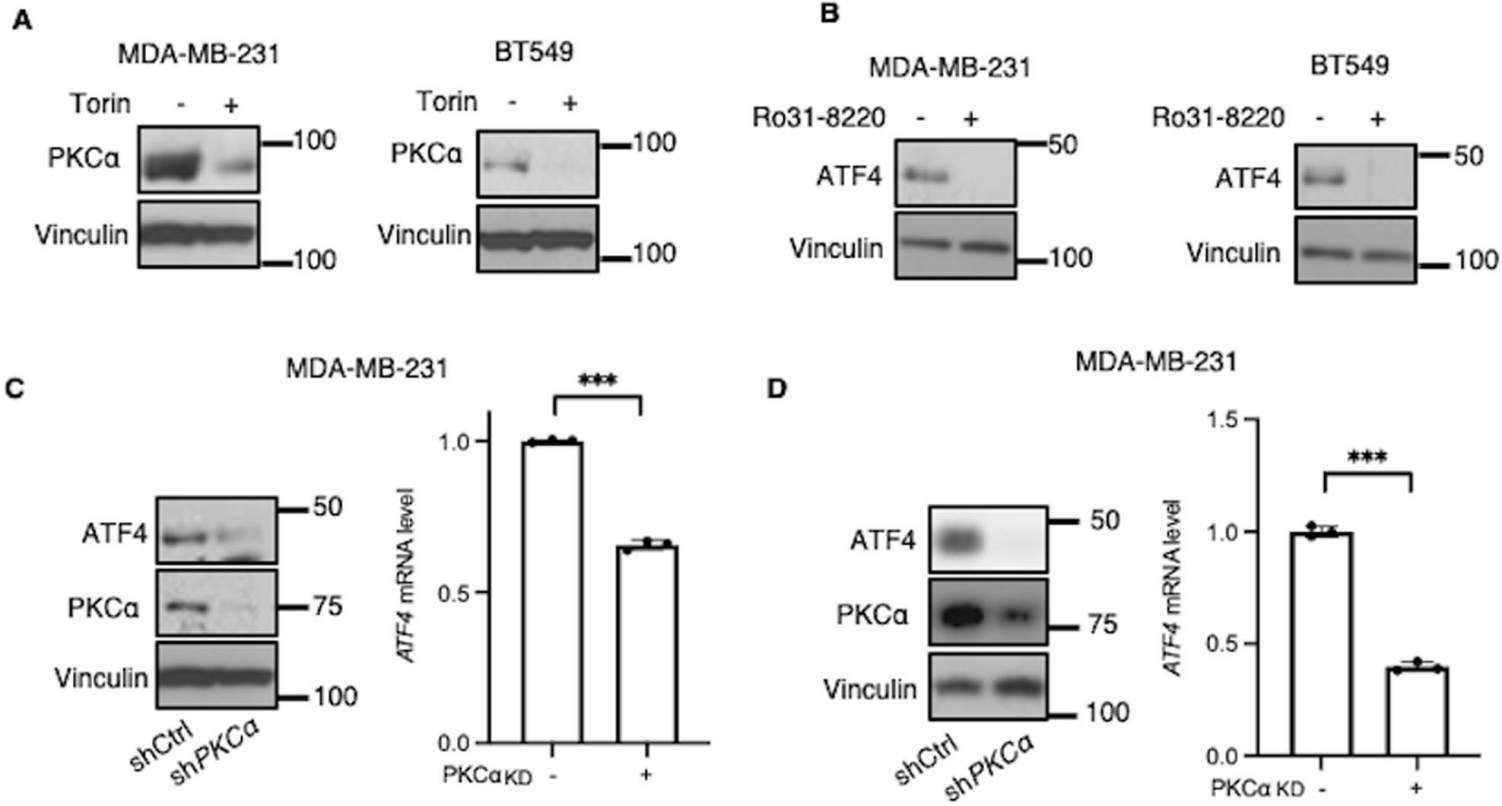
mTORC2 signals to PKC to promote *ATF4* transcription. **A** Western blot analysis of WCL from MDA-MB-231 and BT549 cells treated with CB-839 (1 μM) ± Torin1 (500 nM) for 24 h. Blots are representative of three (MDA-MB-231) or two (BT549) independent experiments. **B** Western blot analysis of WCL from MDA-MB-231 and BT549 cells treated with CB-839 (1 μM) ± Ro31-8220 (5 μM) for 24 h. Blots are representative of two independent experiments. **C** Western blot analysis of WCL and RT-qPCR quantification of *ATF4* mRNA in control and *PKCα* KD MDA-MB-231 cells treated with CB-839 (1 μM) for 24 h. Blots are representative of three independent experiments. qPCR data shown are representative of two independent experiments and expressed as means ± SD for triplicate measurements. **D** Western blot analysis of WCL and RT-qPCR quantification of *ATF4* mRNA in control and *PKCα* KD MDA-MB-231 cells deprived of glutamine for 24 h. Blots are representative of two independent experiments. qPCR data shown are representative of two independent experiments and expressed as means ± SD for triplicate measurements.

Since the mTORC2-dependent increases in ATF4 expression are not dependent upon AKT (Fig. 2C, D), we next examined the role of another mTORC2 substrate, PKCα. To test this possibility, we first assessed the effects of Torin1 on PKCα expression in CB-839 treated cells and found that PKCα protein levels were reduced (Fig. 3A), consistent with reports that mTORC2-mediated phosphorylation of PKC is necessary for its stability [11]. To examine whether PKCα promotes ATF4 expression, we treated MDA-MB-231 and BT549 cells with CB-839 and the PKC inhibitor, Ro31-8220, and found that Ro31-8220 suppressed ATF4 expression (Fig. 3B). We then knocked down *PKCα* in MDA-MB-231 cells and compared ATF4 protein and transcript levels and found that both were decreased in cells treated with CB-839 or deprived of glutamine (Figs. 3C, D, S2F, S2G).

### mTORC2 enhances ATF4 expression via a PKC-Nrf2 axis

**Fig. 4:**
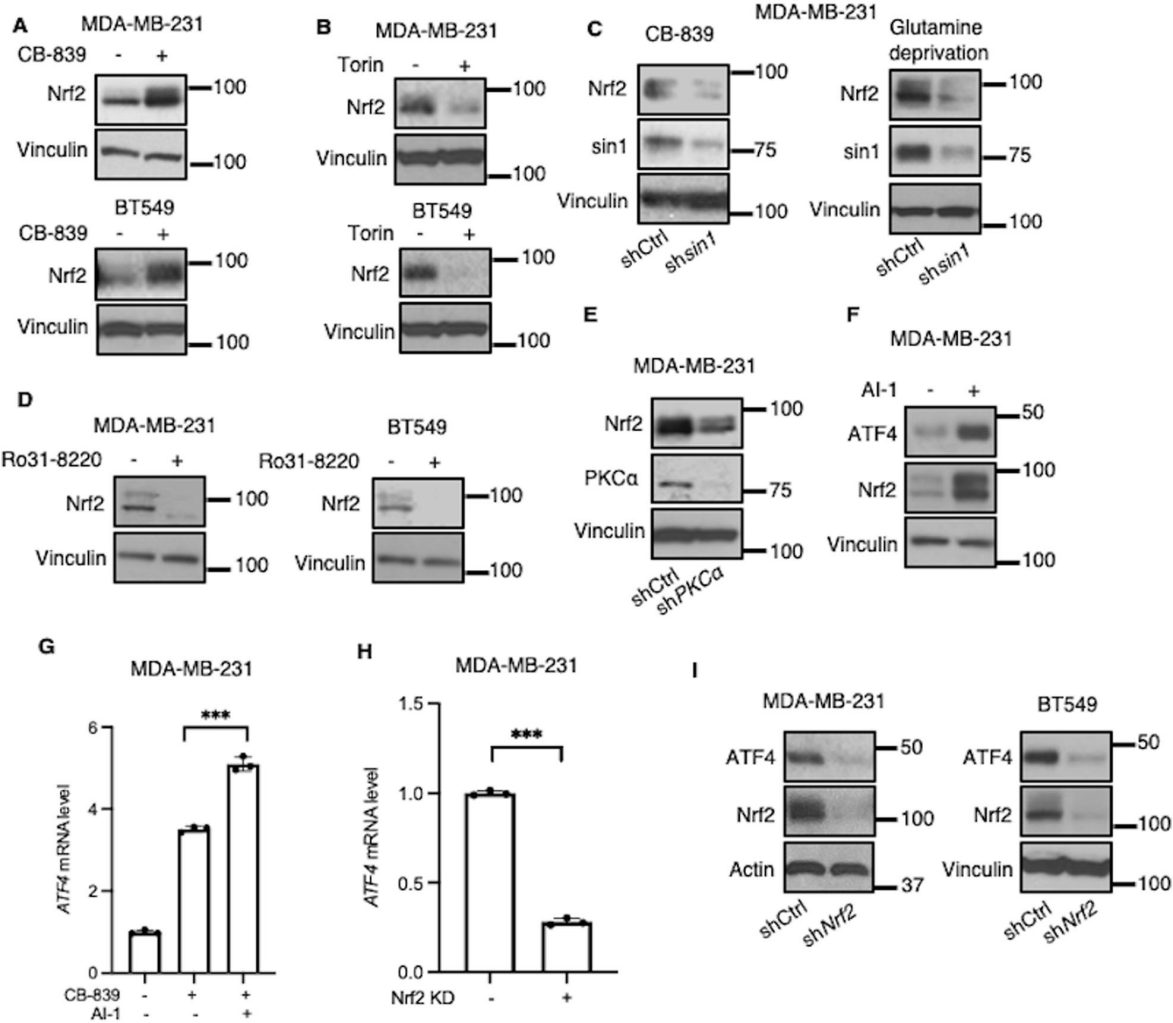
mTORC2-PKC-Nrf2 axis increases ATF4 abundance. **A** Western blot analysis of WCL from MDA-MB-231 and BT549 cells ± CB-839 (1 μM) for 24 h. Blots are representative of two (MDA-MB-231) or three (BT549) independent experiments. **B** Western blot analysis of WCL from MDA-MB-231 and BT549 cells treated with CB-839 (1 μM) ± Torin1 (500 nM) for 24 h. Blots are representative of three independent experiments. **C** Western blot analysis of WCL from control or *sin1* KD MDA-MB-231 cells treated with CB-839 (1 μM) or deprived of glutamine for 24 h. Blots are representative of two independent experiments. **D** Western blot analysis of WCL from MDA-MB-231 and BT549 cells treated with CB-839 (1 μM) ± Ro31-8220 (5 μM) for 24 h. Blots are representative of two independent experiments. **E** Western blot analysis of WCL from control or *PKCα* KD MDA-MB-231 cells treated with CB-839 (1 μM) for 24 h. Blots are representative of two independent experiments. **F** Western blot analysis of WCL from MDA-MB-231 cells treated with CB-839 (1 μM) ± AI-1 (10 mM) for 12 h. Blots are representative of two independent experiments. **G** RT-qPCR quantification of *ATF4* mRNA in MDA-MB-231 cells treated with CB-839 (1 μM) ± AI-1 (10 mM) for 24 h. Data shown are representative of two independent experiments and expressed as means ± SD for triplicate measurements. **H**, **I** RT-qPCR quantification of *ATF4* mRNA (**H**) and Western blot analysis of WCL (**I**) in control and *Nrf2* KD MDA-MB-231 and BT549 cells treated with CB-839 (1 μM) for 24 h. Data shown (**I**) are representative of two independent experiments and expressed as means ± SD for triplicate measurements. Blots are representative of three (MDA-MB-231) or two (BT549) independent experiments.

Nrf2 has been shown to play an important role in maintaining cellular redox homeostasis [34–36], making it an attractive candidate to act in a pathway that leads to increased ATF4 expression [29, 37]. MDA-MB-231 cells that were serum-starved overnight showed increased Nrf2 expression with CB-839 treatment, like what we observed for ATF4 expression. Moreover, Nrf2 expression was also increased in BT549 cells when treated with CB-839 (Fig. 4A). Treatment with Torin1 inhibited CB-839-induced Nrf2 expression (Fig. 4B), consistent with the effects of knocking down *sin1* (Figs. 4C, S3A), whereas rapamycin failed to have an effect (Fig. S3B). PKCα has been shown to phosphorylate Nrf2 and promote its stability [17, 18]. Thus, we hypothesized that mTORC2 stimulates *ATF4* transcription through Nrf2 in a PKCα-dependent manner. To test this idea, we first used Ro31-8220 in cells treated with CB-839 and found that Nrf2 protein levels were strikingly decreased (Fig. 4D). Similarly, knocking down PKCα in cells treated with CB-839 decreased Nrf2 expression (Fig. 4E). In contrast, the Nrf2 activator, AI-1, markedly increased the protein and transcript levels of ATF4 in breast cancer cells either treated with CB-839 or deprived of glutamine (Figs. 4F, G, and S3C). Because Nrf2 is a transcription factor, we examined how knocking down Nrf2 affected the transcript levels of *ATF4*. This was especially relevant as we found in the public CHIP-seq data base (chip-atlas.org), that the Nrf2 antibody bound to the ATF4 promoter (~1 kB from the TSS) in the cardiovascular HAEC cell line. When *Nrf2* was knocked down in cancer cells treated with CB-839 or menadione, there was a reduction in both the transcript and protein levels of ATF4 (Figs. 4H, I and S3D).

### Sirt5 expression is enhanced under conditions of metabolic stress

**Fig. 5:**
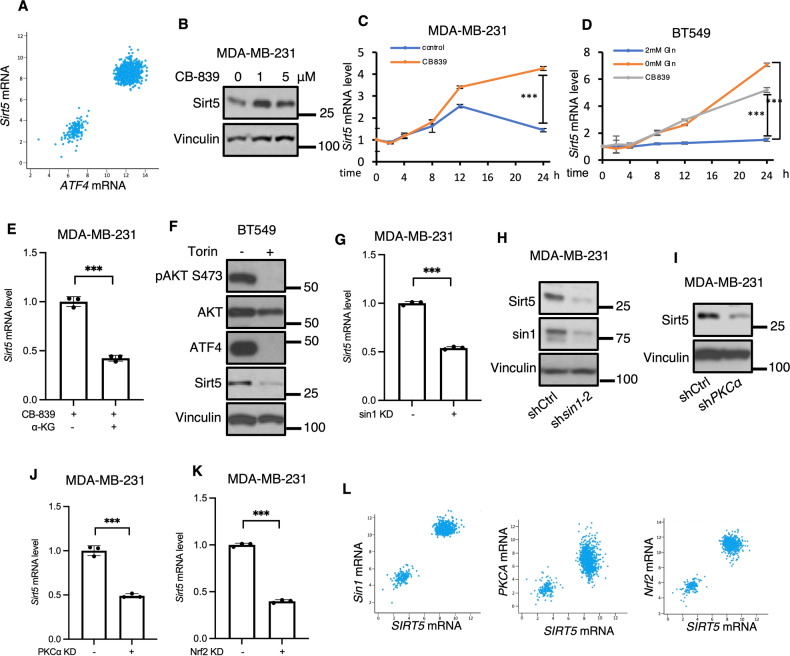
The regulation of Sirt5 expression parallels ATF4. **A** Scatter plots showing *Sirt5* mRNA levels correlates with *ATF4* mRNA levels. Data were collected from TCGA breast cancer dataset. **B** Western blot analysis of WCL of MDA-MB-231 cells ± CB-839 (1 μM) for 24 h. Blots are representative of two independent experiments. **C**, **D** RT-qPCR quantification of *Sirt5* mRNA in MDA-MB-231 and BT549 cells treated ± CB-839 (1 μM) or ± glutamine (2 mM) at the indicated times. Data shown are representative of two independent experiments and expressed as means ± SD for triplicate measurements. **E** RT-qPCR quantification of *Sirt5* mRNA in MDA-MB-231 cells treated with CB-839 (1 μM) ± DM-αKG (2 mM) for 24 h. Data shown are representative of two independent experiments and expressed as means ± SD for triplicate measurements. **F** Western blot analysis of WCL from BT549 cells treated with CB-839 (1 μM) ± Torin1 (500 nM) for 4 h. Blots are representative of two independent experiments. **G** RT-qPCR quantification of *Sirt5* mRNA and Western blot analysis of WCL in control and *sin1* KD MDA-MB-231 cells treated with menadione (10 μM) for 24 h. Data shown are representative of two independent experiments and expressed as means ± SD for triplicate measurements. **H** Western blot analysis of WCL from control or *sin1* KD MDA-MB-231 cells treated with CB-839 (1 μM) for 24 h. Blots are representative of two independent experiments. **I**, **J** Western blot analysis of WCL and RT-qPCR quantification of *Sirt5* mRNA in control and *PKCα* KD MDA-MB-231 cells treated with CB-839 (1 μM) for 24 h. Blots are representative of two independent experiments. qPCR data shown are representative of two independent experiments and expressed as means ± SD for triplicate measurements. **K** RT-qPCR quantification of *Sirt5* mRNA in control and *Nrf2* KD MDA-MB-231 cells treated with menadione (10 μM) for 24 h. Data shown are representative of two independent experiments and expressed as means ± SD for triplicate measurements. **L** Scatter plots show Sirt5 mRNA levels correlates with *sin1*, *PKCA* and *NRF2* mRNA levels in breast cancer. Data are from TCGA breast cancer dataset.

The results described above point to an mTORC2-directed signaling pathway which responds to perturbations in glutamine metabolism and oxidative stress with the prolonged expression of *ATF4* via transcriptional regulation. As the expression of ATF4 provides a protective benefit in breast cancer cells challenged with metabolic stress (Fig. 1H), we next wanted to identify transcriptional targets of ATF4 that play a functional role in this stress response. This led us to Sirt5, as we found a positive correlation between *ATF4* and *Sirt5* expression when analyzing data from The Cancer Genome Atlas (TCGA) breast cancer data set (3380 samples, Fig. 5A).

Because Sirt5 supports glutamine metabolism in cancer cells by protecting GAC from degradation, we examined whether Sirt5 expression was increased in a manner like ATF4 in response to metabolic stress. CB-839 treatment for 24 h resulted in increased Sirt5 (Fig. 5B), similar to ATF4 (Fig. 2B). *Sirt5* mRNA levels were also enhanced when either MDA-MB-231 cells or BT549 cells were treated with CB-839 or deprived of glutamine. Increases in *Sirt5* occurred over 24 h of CB-839 treatment or glutamine withdrawal, compared to serum deprivation (Fig. 5C, D). Oxidative stress also enhanced *Sirt5* expression, as evidenced when either BT549 or MDA-MB-231 cells were treated with menadione (Fig. S4A). Additionally, since increases in *Sirt5* occurred when cancer cells were prevented from undergoing glutaminolysis, we tested whether increases in *Sir*t5 expression accompanying these stresses could be suppressed by supplementation with DM-αKG and found that to be the case (Fig. 5E). Additionally, treatment with ActD significantly blocked an increase of *Sirt5* that accompanies CB-839 treatment and glutamine depletion (Fig. S4B, C).

### Stress-induced mTORC2 signaling promotes Sirt5 transcription

Given the correlation between the expression patterns of Sirt5 and ATF4 in response to metabolic stress, we wanted to determine if the stress-induced mTORC2 signaling pathway outlined above was responsible for the observed increases in Sirt5 expression. Indeed, we found that Torin1 treatment decreased Sirt5 expression in CB-839 treated BT549 cells (Fig. 5F). Knocking down *sin1* also decreased *Sirt5* transcript and protein levels in MDA-MB-231 cells treated with menadione, CB-839 or deprived of glutamine (Figs. 5G, H, S4D), as did knocking down *PKCα* (Figs. 5I, J, S4E, F) or treatment with Ro31-8220 (Fig. S4G). Genetic silencing of *Nrf2* (Fig. 5K) also reduced *Sirt5* transcript levels in MDA-MB-231 cells treated with menadione, and TCGA data showed that *Sirt5* positively correlates with *sin1* (Spearman = 0.16; Pearson = 0.88), *PKCA* (Spearman = −0.04; Pearson = 0.50) and *Nrf2* (Spearman = −0.01; Pearson = 0.81), (Fig. 5L). Together, these data demonstrate that *Sirt5* is a transcriptional target of a stress-induced mTORC2 signaling pathway.

### Sirt5 is a transcriptional target of ATF4

**Fig. 6:**
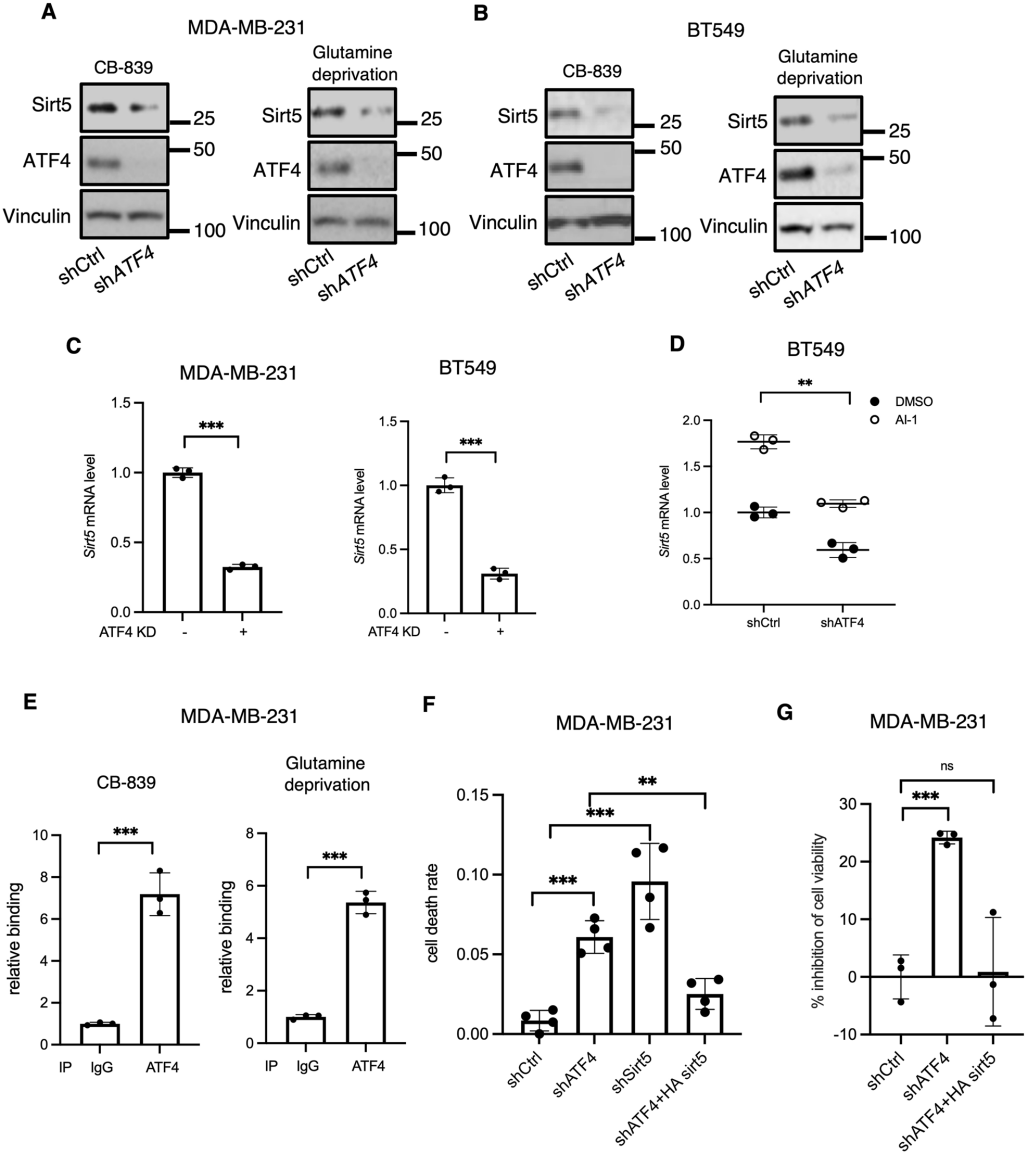
Sirt5 is a transcriptional target of ATF4 necessary for survival. **A** Western blot analysis of WCL from control or *ATF4* KD MDA-MB-231 cells treated with CB-839 (1 μM) for 8 h or without glutamine for 24 h. Blots are representative of three independent experiments. **B** Western blot analysis of WCL from control or *ATF4* KD BT549 cells treated with CB-839 (1 μM) or without glutamine for 24 h. Blots are representative of two independent experiments. **C** RT-qPCR quantification of *Sirt5* mRNA in control and *ATF4* KD MDA-MB-231 and BT549 cells treated with CB-839 (1 μM) for 8 h (MDA-MB-231) or 24 h (BT549). Data shown are representative of two independent experiments and expressed as means ± SD for triplicate measurements. **D** RT-qPCR quantification of *Sirt5* and *ATF4* mRNA in control and *ATF4* KD BT549 cells treated with CB-839 (1 μM) ± AI-1 (10 mM) for 24 h. Data shown are representative of two independent experiments and expressed as means ± SD for triplicate measurements. Differences were analyzed with two-way AVOVA. **E** ChIP analysis of ATF4 binding to the *Sirt5* promoter in MDA-MB-231 cells treated CB-839 (1 μM) or without glutamine (2% dialyzed FBS) for 8 h. Data shown are representative of two independent experiments and expressed as means ± SD for triplicate measurements. **F** The percentage of TUNEL positive cells (*n* = 4). Data shown are representative of two independent experiments. **G** CCK8 assay showing the percent inhibition of cell viability for control, ATF4 knockdown and ATF4 knockdown cells with the ectopic expression of Sirt5. MDA-MB-231 cells were cultured in glutamine free medium for 48 h. Data shown are representative of two independent experiments and expressed as means ± SD for triplicate measurements.

We next set out to establish that *Sirt5* is a transcriptional target of ATF4 by examining the effects of knocking down its expression in breast cancer cells undergoing metabolic stress. When *ATF4* knockdown and control MDA-MD-231 or MDA-MB-468 cells were treated with CB-839, we found that depleting ATF4 significantly reduced Sirt5 protein levels (Figs. 6A, S5A). Knocking down *ATF4* also led to a decrease in Sirt5 protein levels in glutamine-deprived cells (Fig. 6A). The same was true when knocking down *ATF4* in BT549 breast cancer cells treated with CB-839 or deprived of glutamine (Fig. 6B). Likewise, the genetic silencing of *ATF4* reduced *Sirt5* transcript levels in MDA-MB-231 cells and in BT549 cells (Figs. 6C, S5B) treated with CB-839, as was the case when they were deprived of glutamine (Fig. S5C) or treated with menadione (Fig. S5D). Treatment with AI-1 increased levels of both *Sirt5* and *ATF4* in BT549 cells treated with CB-839 (Fig. 6D). However, when *ATF4* was knocked down under these conditions, AI-1 treatment did not increase *Sirt5* transcript levels (Fig. 6D). These findings indicate that *Sirt5* is downstream of ATF4.

We next examined whether ATF4 binds directly to the *Sirt5* promoter. The promoter region for the human *Sirt5* gene was analyzed -2000 bp relative to the transcription start site (TSS), and several ATF4 binding sites were identified using the PATCH resource. A close match was found to the consensus ATF4-binding motif (TGATGXAAX) at position −1087 bp, relative to the TSS of the *Sirt5* promoter. We then carried out chromatin immunoprecipitations (ChIPs) to test ATF4 binding to the *Sirt5* promoter. MDA-MB-231 breast cancer cells were treated with CB-839 for 8 h; cross-linked chromatin was then digested to a length of ~150–900 bp, and an antibody against ATF4 was used to precipitate ATF4/DNA complexes. A parallel immunoprecipitation was carried out using IgG. Protein-DNA cross-links were then reversed, and qPCR was performed using primers designed to amplify the identified ATF4-binding motif. This yielded a markedly stronger signal from the ATF4 ChIP relative to the IgG ChIP. (Fig. 6E). Glutamine deprivation also increased the binding of ATF4 to the *Sirt5* promoter (Fig. 6E), thus demonstrating that *Sirt5* transcription is directly promoted by ATF4 in response to metabolic stress.

### ATF4-Sirt5 axis provides a survival response to metabolic stress

Glutaminolysis is important for the survival of cancer cells when challenged by different types of stress, and Sirt5 plays a key role in providing protection against these challenges through its ability to stabilize the cellular expression of GAC [6]. Thus as expected, knocking down *Sirt5* in MDA-MB-231 cells cultured under conditions of glutamine deprivation resulted in an increased rate of cell death, as read-out by the TUNEL assay (Figs. 6F and S5E). This was also the case when knocking down *ATF4*. However, when Sirt5 was ectopically overexpressed in cells depleted of ATF4, cell survival was markedly increased (Figs. 6F and S5E). Similarly, knocking down *ATF4* reduced cell viability under glutamine deprived conditions, while the ectopic expression of Sirt5 maintained cell viability in ATF4 knockdown cells (Fig. 6G), indicating that *Sirt5* is a physiologically relevant target of ATF4 to promote cancer cell survival in the face of metabolic stress.

### Broader implications for the mTORC2-PKC-Nrf2-ATF4-Sirt5 axis in cancer

As mentioned above, the TCGA analysis of the breast cancer data base showed a very high correlation between *Sirt5* expression and the expression of genes encoding proteins involved in the mTORC2-PKC-Nrf2-ATF4 signaling pathway described herein (Fig. 5A, L). However, we also observed a striking bimodal distribution which represents breast cancer cells that express these different genes in either relatively low or high abundance. We suspected that this bimodal distribution might be consistent with the ability of cancer cells to activate the mTORC2-PKC-Nrf2-ATF4-Sirt5 pathway in response to metabolic stress. Thus, we were interested in seeing whether this bimodal signature might have predictive value in identifying cancer cells where this signaling pathway is triggered to promote survival.

**Fig. 7:**
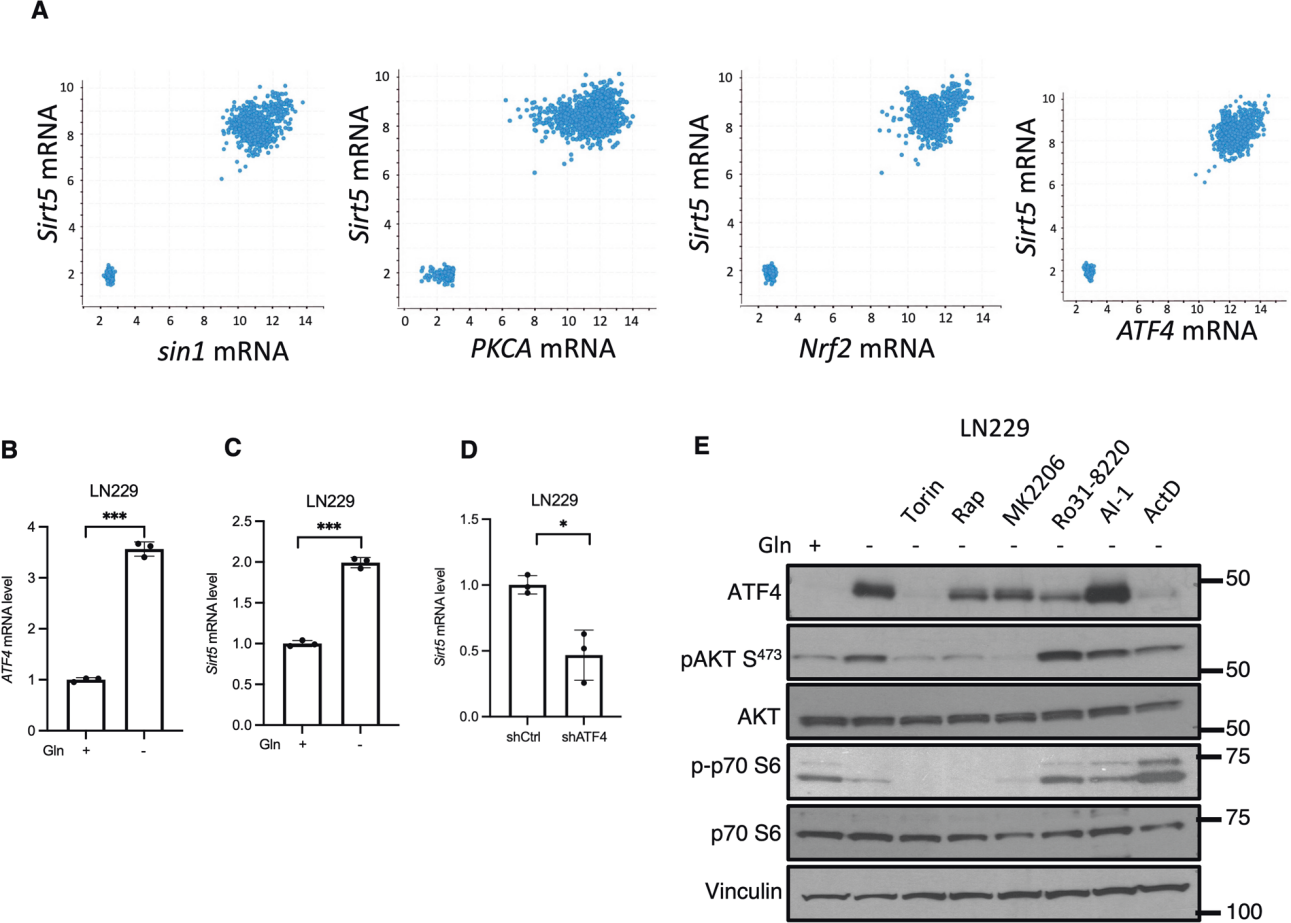
Evidence for the mTORC2-PKC-Nrf2-ATF4-Sirt5 pathway in brain cancer. **A** Scatter plots showing that Sirt5 mRNA levels correlate with *sin1* (Spearman: 0.27 Pearson: 0.96), *PKCA* (Spearman: 0.37 Pearson: 0.92), *NRF2* (Spearman: 0.23 Pearson: 0.96) and *ATF4* (Spearman: 0.38 Pearson: 0.97) mRNA levels in brain cancer. Data are from the TGCA brain cancer dataset. **B** RT-qPCR quantification of *ATF4* mRNA in LN229 cells cultured ± glutamine in serum free medium for 24 h. Data shown are representative of two independent experiments and expressed as means ± SD for triplicate measurements. **C** RT-qPCR quantification of *Sirt5* mRNA in LN229 cells cultured ± glutamine in serum free medium for 24 h. Data shown are representative of two independent experiments and expressed as means ± SD for triplicate measurements. **D** RT-qPCR quantification of *Sirt5* mRNA in control and *ATF4* KD LN229 cells cultured in glutamine free medium for 24 h. Data shown are representative of two independent experiments and expressed as means ± SD for triplicate measurements. **E** Western blot analysis of WCL from LN229 cells treated with Torin1 (500 nM), Rapamycin (50 nM), MK2206 (5 μM), Ro31-8220 (1 μM), AI-1 (10 mM) or ActD (1 μg/mL) in serum/glutamine free medium for 24 h. Blots are representative of two independent experiments.

To examine this possibility, we extended the TGCA analysis by analyzing the expression correlation of the pathway genes in 5 additional cancer types: lung (330 samples), lymphoid (310 samples), ovarian and fallopian tube (1047 samples), pancreatic (356 samples), and brain (1894 samples). The first four cancer types did not display any significant correlation amongst the expression of the genes encoding the mTORC2-PKC-Nrf2-ATF4-Sirt5 signaling pathway (Fig. S6A–D). In contrast, the expression of these genes was highly correlated with Sirt5 in brain cancer (Pearson values: *sin1* 0.96; *PRCA* 0.92; *Nrf2* 0.96; *ATF4* 0.97) and demonstrated the characteristic bimodal distribution observed in breast cancer (Fig. 7A).

To follow up on the similarity between pathway gene expression patterns in breast cancer and brain cancer, we examined how the expression of *ATF4* and/or *Sirt5* was affected in the glioblastoma cell line LN229 upon glutamine withdrawal. qPCR analysis revealed that like the case in triple negative breast cancer, glutamine withdrawal resulted in increases in *ATF4* and *Sirt5* mRNA expression (Fig. 7B, C), and that *Sirt5* mRNA transcript levels in cells deprived of glutamine were diminished upon the knockdown of *ATF4* (Fig. 7D). Additionally, the protein expression of ATF4 was significantly enhanced upon glutamine withdrawal, or when treating cells with the Nrf2 activator, AI-1, but this was completely reversed by treatment with Torin and ActD, and largely reduced by the PKC inhibitor Ro31-8220 (Fig. 7E). In contrast to the triple negative breast cancer cell lines, we also see a partial reduction in ATF4 expression using rapamycin and MK2206 in LN229 cells, suggesting the ability of additional signaling pathways to contribute to this stress response in these cells. In summary, these observations are consistent with the activation of the mTORC2-PKC-Nrf2-ATF4-Sirt5 pathway in LN229 glioblastoma cells upon nutrient deprivation, as well as demonstrate the potential of utilizing the bimodal RNA transcript signature that we observe to identify cancer cells where this signaling pathway may play an essential role in their survival.

## Discussion

**Fig. 8:**
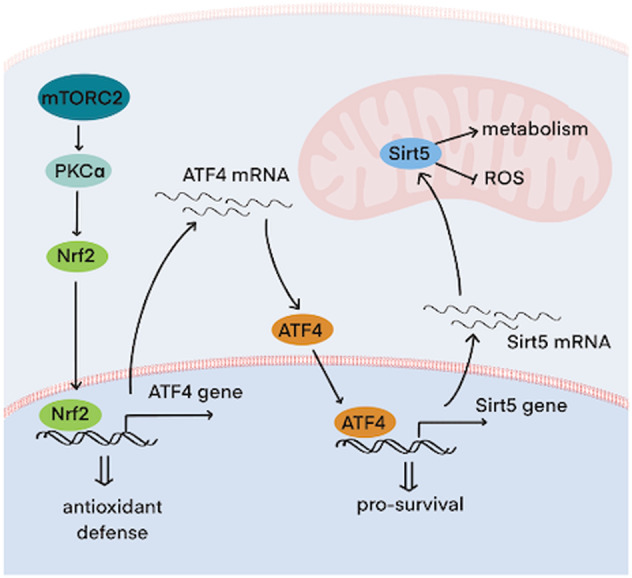
Model for a stressed-induced mTORC2 signaling pathway targeting ATF4/Sirt5 in cell survival. mTORC2 senses cellular stresses arising from glutamine deprivation or an acute inhibitionof glutamine metabolism. Upon its phosphorylation by mTORC2, PKC targets Nrf2 which triggers sustained *ATF4* and *Sirt5* transcription and subsequent protein production to promote cell survival in the face of metabolic and oxidative stress.

We show that mTORC2 enhances *ATF4* expression and identify *Sirt5* as an ATF4 transcriptional target which is necessary to promote breast cancer survival in response to metabolic stress. In addition to the canonical regulation of ATF4 translation by the ISR pathway, we find that mTORC2 activation increases *ATF4* transcription which is critical for sustaining ATF4 pools during pro-longed exposure of cancer cells to stress. mTORC2 signals for an increase in *ATF4* transcription through a PKCα-Nrf2 axis, highlighting an AKT-independent survival pathway. The expression of *Sirt5*, a key transcriptional target of this pathway, is directly increased through the regulatory actions of ATF4 and is essential for cell survival when glutamine metabolism is limited (Fig. 8). Thus, these findings identify a mechanism by which mTORC2 serves as a metabolic stress sensor to facilitate the ability of cancer cells to overcome challenges caused by ROS elevation or compromised glutamine metabolism.

Previous studies found that ATF4 expression is important for cancer cell proliferation and survival during nutrient deprivation, including glutamine withdrawal, and consequently numerous metabolic enzymes targeted by ATF4 have been identified [28–30, 38–41]. However, these studies have mainly emphasized the importance of ATF4 protein expression, either through general control nonderepressible 2 (GCN2)-dependent translation [28] or p62-dependent degradation [42], and it is still unclear how *ATF4* gene expression is increased in TNBC patients. Given that the tumor microenvironment exposes cancer cells to sustained conditions of stress, together with the fact that ATF4 protein half-life is only 30 minutes [42], elevated *ATF4* transcription is essential for cancer cells to preserve an *ATF4* mRNA reservoir and ensure continuous ATF4 protein translation. Here, we describe a unique signaling pathway that triggers the upregulation of *ATF4* expression in breast cancers challenged with metabolic stress. Notably, while most known metabolic pathways regulated by mTORC2 are dependent upon AKT, we now show that mTORC2 activates PKCα/Nrf2 to signal an increase in *ATF4* message levels, highlighting a new role for PKCα in metabolic and redox hemostasis regulation.

The sirtuin family members, Sirt3, Sirt4, and Sirt5, have been shown to have important functions in mitochondrial metabolism. While Sirt3 and Sirt4 have been identified as tumor suppressors [43–45], Sirt5 has been reported to be a tumor promoter in various types of cancer [6, 46–51]. In addition to its regulation of several TCA cycle enzymes [4, 52, 53], Sirt5 has a general role in glutamine metabolism. Sirt5 activates GDH [50] and stabilizes GAC [6], two key enzymes in glutaminolysis, and detoxifies ammonia produced from the GAC-catalyzed hydrolysis of glutamine by activating CPS1 [51]. Many cancer cells increase Sirt5 expression; however, the underlying mechanism has remained elusive. Here, we now demonstrate that *Sirt5* expression is increased by an mTORC2-ATF4 signaling pathway in response to metabolic stress. Several Sirt5-activated substrates are essential for redox balance, underscoring the importance of elevated Sirt5 expression in response to the oxidative stress.

### mTORC2 and Sirt5 as potential therapeutic targets

Clinical trials have shown that mTOR inhibitors can improve ER^+^ or HER2^+^ breast cancer patient outcome but failed in TNBC patients, possibly due to a distinct activation of mTOR [52]. mTORC1 is known to transmit its signals to ATF4 upon growth factor stimulation [38], and promote *ATF4* mRNA translation [53]. Distinctly, under conditions of metabolic and oxidative stress in triple negative breast cancers, we found that mTORC2 activation leads to elevated ATF4 expression, overall suggesting that ATF4 could serve as a biomarker for treatment with mTOR inhibitors in some cancers. Additionally, our TCGA analysis using different cancer types further suggests that some, but not all, cancers might utilize the mTORC2-PKC-Nrf2-ATF4-Sirt5 pathway to promote cell survival in response to stress, based on the relationship between the expression of genes encoding proteins in this signaling pathway. In particular, we found that the expression of pathway genes in brain cancer, like breast cancer, is highly correlated with distinct populations of low and high mRNA-expressing cancers, but this was not the case for lung, lymphoid, ovarian or pancreatic cancer. We also found evidence that this signaling pathway can be activated in glioblastoma cells in response to metabolic stress. Therefore, further investigation into the predictive value of the relationship between pathway genes is warranted as it may provide a mechanism to identify cancers that could be susceptible to therapeutic strategies targeting components of this pathway.

An important question concerns how mTORC2 senses decreased glutamine metabolism. The Fingar group showed that AMPK activates mTORC2 through direct phosphorylation during acute energetic stress [54], and in response to oxidative stress, the Rit GTPase interacts with sin1 to activate mTORC2 [55]. In glutamine-depleted lung cancer cells, Sestrin2 is induced by glutamine deprivation to promote mTORC2 activity by interrupting the interaction between 14-3-3 and Rictor [56]. Another intriguing possibility is that some metabolic intermediates might interact with mTORC2 directly, or that an upstream stress sensor activates mTORC2. Finally, a broader question of interest is how ubiquitous of a role does this mTORC2-ATF4-Sirt5 pathway play in the ability of cancer cells to adapt and survive different challenges imposed by their microenvironment.

## Materials and methods

### Cell culture and media

Breast cancer cell lines MDA-MB-231, BT549, MDA-MB-468 and HEK 293 T cells were obtained from the American Type Cell Culture Collection (ATCC, US). All breast cancer cell lines were maintained at 37 °C, 5% CO2 atmosphere, in RPMI 1640 medium containing 2 mM glutamine (Gibco, US, MA) and supplemented with 10% FBS (Gibco). HEK 293 T cells were cultured in DMEM (Gibco) supplemented with 10% FBS (Gibco). For glutamine-withdrawal experiments, glutamine-free RPMI 1640 medium (Gibco) was used. For signaling experiments, serum free RPMI was used. Cell lines were periodically tested for Mycoplasma contamination.

### Antibodies and reagents

Antibodies recognizing the following proteins were purchased from Cell Signaling Technology (US, MA): HA-tag (cat. no. 3724) used at 1:2000, Vinculin (cat. no. 13901) used at 1:10,000, Sirt5 (cat. no. 8782) used at 1:1000, ATF4 (cat. no. 11815) used at 1:1000, phospho-Akt (S473) used at 1:1000, AKT (cat. no. 9272) used at 1:1000, beta-actin (cat. no. 3700) used at 1:1000, caspase3 (cat. no. 9662) used at 1:1000, PKC alpha (cat. no. 2056) used at 1:1000, phospho-p70 S6 kinase (T389) (cat. no. 9205) used at 1:1000, and p70 S6 kinase (cat. no. 9202) used at 1:1000. The antibody recognizing Nrf2 was purchased from Proteintech (cat. no. 16396-1-AP, US, IL) and used at 1:5000. The antibody recognizing sin1 was purchased from Sigma (cat. no. 05–1044, US, MA) and used at 1:1000. Secondary antibodies used, as appropriate, were Cell Signaling Technology anti-rabbit IgG, HRP-linked (cat. no. 7074) or anti-mouse IgG, HRP-linked (cat. no. 7076). CB-839, Torin1, MK2206 were purchased from Selleckchem (US, TX). All other reagents were purchased from Sigma-Aldrich unless otherwise stated.

### Lentivirus system for knockdown and ectopic expression

Short hairpin RNA (shRNA)-encoding plasmids for knocking down *ATF4*, *sin1*, *PKCα* and *Sirt5* were from Sigma (TRCN0000013573 and TRCN0000013574 for *ATF4*, TRCN0000003153 and TRCN0000003151 for *sin1*, TRCN0000195250 and TRCN0000001690 for *PKCα*, TRCN0000018544 for Sirt5). Note, the first of each shRNA pair was used for experiments in the figures presented in the main body of the Results, while second of each pair was used in experiments presented in the Supplementary Information. For ectopic expression, *Sirt5* cDNA was subcloned into plasmid pSin-EF2-Oct4-Pur. To generate viruses, the desired plasmid construct (6 μg) was mixed with virus package plasmids pCMV.d8.2 (4 μg) and pMDG (2 μg) in 400 μl DMEM medium, followed by the addition of 30 μl of 1 mg/ml PEI (polyethylenimine MW25K, Polysciences Inc, US, PA). The mix was incubated at room temperature for 15 min and then added to HEK 293 T cells at 80% confluence in a 10 cm dish, containing 8 ml of complete DMEM medium. After overnight incubation, the medium was replaced with 15 ml complete DMEM medium. Virus-containing supernatant was harvested at 24, 48, and 72 h post transfection. Individual harvests were pooled and filtered through a 0.45 μm PES filter, aliquoted and stored at −80 °C.

### Western blot analysis

Whole-cell lysates were prepared in lysis buffer (50 mM HEPES pH 8.0, 150 mM NaCl, 1 mM Na_3_VO_4_, 25 mM NaF, 1% (v/v) Triton X-100, 1 mM MgCl_2_, 50 mM β-glycerophosphate, 30 mgml^−1^ leupeptin, 5 mgml^−1^ aprotinin). Protein concentration was determined by Bradford assay (Bio-Rad, US, CA), and lysate proteins denatured by boiling for 3 min in reducing SDS-sample buffer. Lysate proteins (30 μg total protein/lane) were then resolved on Novex 4–20% Tris-glycine mini (Life Technologies) and transferred to PVDF Transfer membranes (Thermo Scientific). Membranes were blocked in 25% (v/v) non-fat dry milk in tris-buffered saline and tween 20 (TBST) for 1 h at room temperature and probed overnight at 4 °C in primary antibody solution (manufacturer recommended concentration) in TBST. They were then washed in TBST and incubated in TBST containing appropriate secondary antibody at the manufacturer’s recommended concentration for 1 h. Finally, the membranes were washed in TBST, and bands imaged using Western Lightning Plus-ECL (PerkinElmer, US, MA) and High sensitivity autoradiography film (Thomas Scientific). The sample size for Western blotting experiments is *n* = 1 and individual replicates are 2–3 experiments.

### Real-time PCR analysis

Total RNA was isolated from cells using the Direct-zol RNA Microprep (Zymo Research, US, CA), and a cDNA library prepared by reverse transcription using the SuperScript III first-strand synthesis system (Life Technologies). Quantitative real-time PCR analysis was carried out using cDNA as a template, specific primers and iTaq Universal SYBR Green Supermix (Bio-Rad). Reactions were performed using the 7500 fast real-time PCR system (Applied Biosystems, US, MA). Primer sets used were (5‘→3‘): *ATF4* (ATF4-F, GGCCAAGCACTTCAAACCTC and *ATF4*-R, GAGAAGGCATCCTCCTTGCTG) and *Sirt5* (Sirt5-F, TCGCCCACTGTGATT TATGTC, and *Sirt5*-R, ACCTGAATCTGTTCGTAGCTG). Primers for actin (*actin*-F, CATGTA CGTTGCTATCCAGGC, and *actin*-R, CTCCTTAATGTCACGCACGAT) or *18* *S rRNA* (18 S rRNA-F, CGGCGACGACCCATTCGAAC, and *18* *S rRNA*-R, GAATCGAACCCTGATTCCCCGTC) were used as endogenous controls. The sample size for qPCR experiments is n = 3 and each individual experiment was replicated 2–3 times.

### Chromatin immunoprecipitation

Chromatin immunoprecipitations were performed using the SimpleChIP enzymatic chromatin IP kit (Cell Signaling Technology), following the manufacturer’s instructions. MDA-MB-231 cells (4*15 cm dishes at ~85% confluency) were used as the source of chromatin. Analysis following chromatin digestion showed that DNA was digested to fragments of the desired size (150–900 bp, equivalent to 1–5 nucleosomes). An antibody against endogenous ATF4 (Cell Signaling Technology, 11815) was used to immunoprecipitate complexes containing ATF4. Following reversal of protein–DNA complexes and purification of DNA, RT–PCR was carried out as described above but using the purified DNA as a template. One primer set was designed to amplify 156 bp fragments containing the ATF4 binding site of the Sirt5 promoter at position −1080 bp relative to the TSS. Forward primer: 5‘-GATAACAGTACCTATTT -3‘; Reverse primer: 5‘-CCTCTCTTT TGATTGGCGATTAGGG-3‘. The sample size for ChIP experiments is *n* = 3 and the individual experiment was replicated 2 times.

### DCFDA assay

MBD-MA-231 cells were seeded on a black, clear bottom 96 well microplate with 10000 per well. After overnight adhesion, cells were washed with PBS and treated with CB-839 in phenol red free RPMI. After 24 h, 2X DCFDA solution in PBS was added for 20 μM final concentration. Fluorescence was measured after 45 min incubation with plate reader at Ex/Em = 485/535 nm (TECAN, SPARK). The sample size for the DCFDA assay is *n* = 3 and each individual experiment was replicated 2 times.

### Viability assays

#### CCK8 assay

The CCK8 assay kit (Dojindo, SKU: CK04) was used to measure cell viability.10000 cells were seeded on 96 well plates overnight. The next day, cells were washed with PBS three times and incubated in serum/gultamine-free medium for 48 h. The CCK8 reagent was added according to the manufacturer’s protocol, and OD 490 was obtained from the plate reader. The sample size for CCK8 experiments is *n* = 3, and the individual experiment was replicated 2 times.

#### TUNEL assay

The In situ cell death detection kit (Roche, 11684795910) was used to perform TUNEL assays. 10000 cells were seeded on 16 well chamber slides overnight. The next day, cells were washed with PBS three times and incubated in serum/gultamine-free medium for 48 h. TUNEL staining was performed according to the manufacturer’s protocol. Images were obtained from the fluorescent microscope. The sample size for the TUNEL assay was *n* = 4, and the individual experiment was replicated 2 times.

### TCGA data

The Cancer Genome Atlas (TCGA) Breast Invasive Carcinoma (TCGA, provisional), Brain Cancer, Lung Cancer, Lymphoid, and Ovary/Fallopian Tube Cancer and Pancreatic Cancer data sets were accessed, all data were analyzed, and correlation plots were prepared using the cBioportal suite of tools (www.cbioportal.org). mRNA (microarray) sample data from complete data sets were used in the analysis.

### Statistical analyses

All differences were analyzed with Student’s t-test or two-way AVOVA. In experiments where the sample size was >1, all data points are provided as scatter within bar graphs, and s.d. is calculated for the error bars. All sample sizes chosen are standard for the experiment type.

## Supplementary information

Supplementart Materials

Uncropped Western Blot

## Data Availability

All data are available in the main text or the supplementary materials.
