# Supplementary material for: Elucidation of an mTORC2-PKC-NRF2 pathway that sustains the ATF4 stress response and identification of Sirt5 as a key ATF4 effector
Source: Cell Death Discov. 2022 Aug 13;8:357. doi: 10.1038/s41420-022-01156-5 (PMC9376072; doi:10.1038/s41420-022-01156-5)
Supplement: Supplementary file 1 — Supplementart Materials [file 41420_2022_1156_MOESM1_ESM.docx]

Supplementary Information for

Elucidation of an mTORC2-PKC-NRF2 pathway that sustains the ATF4 stress response and identification of Sirt5 as a key ATF4 effector

**Authors:** Ruizhi Li ^1^, Kristin F. Wilson ^1^, Richard A. Cerione^1,2^*

**Affiliations:**

^1^ Department of Molecular Medicine, Cornell University, Ithaca, NY 14853, USA

^2^ Department of Chemistry and Chemical Biology, Cornell University, Ithaca, NY 14853,

*Corresponding author. Email: rac1@cornell.edu

**This PDF file includes:**

Figs. S1 to S6

**
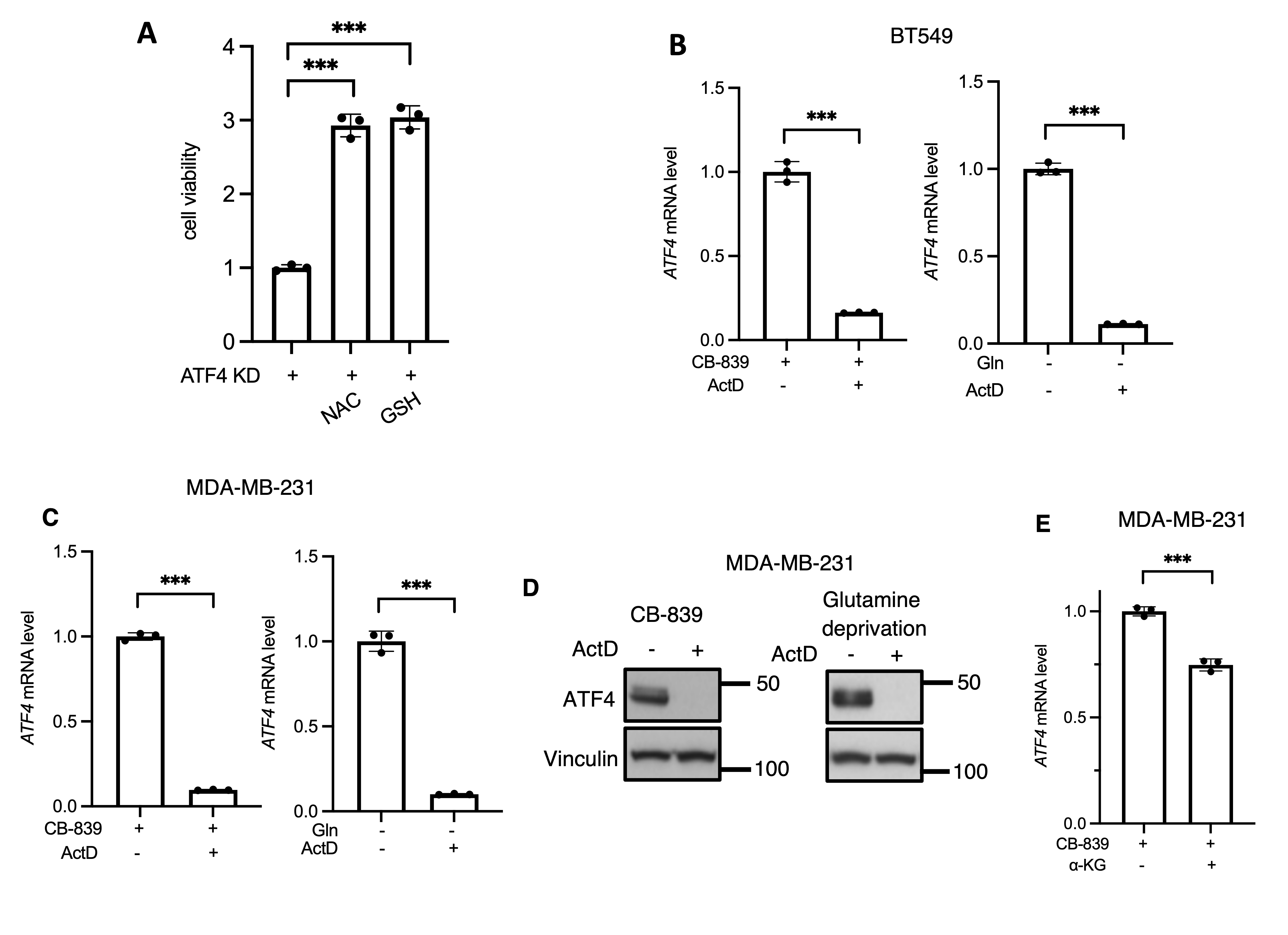
**

**Fig. S1. ATF4 gene expression is the target of oxidative stress signaling in breast cancer.**

**A** CCK8 assay showing the relative cell viability in ATF4 knockdown cells treated with antioxidant NAC or GSH. MDA-MB-231 cells were cultured in glutamine free medium for 48h. Data shown are representative of two independent experiments and expressed as means ± SD for triplicate measurements.

**B, C** RT-qPCR quantification of *ATF4* mRNA in MDA-MB-231 and BT549 cells treated with CB 839 (1 μM) or without glutamine ± ActD (1 μg/mL) for 24 h. Data shown are representative of two independent experiments and expressed as means ± SD for triplicate measurements.

**D** Western blot analysis of WCL from MDA-MB-231 cells treated with CB 839 (1 μM) or without glutamine ± ActD (1 μg/mL) for 24 h. Blots are representative of two independent experiments.

**E** RT-qPCR quantification of *ATF4* mRNA in MDA-MB-231 cells treated with CB 839 (1 μM) ± DM-αKG (2 mM) for 24 h. Data shown are representative of two independent experiments and expressed as means ± SD for triplicate measurements. *p<0.05, **p<0.01 and ***p<0.001 by Student’s t-test.


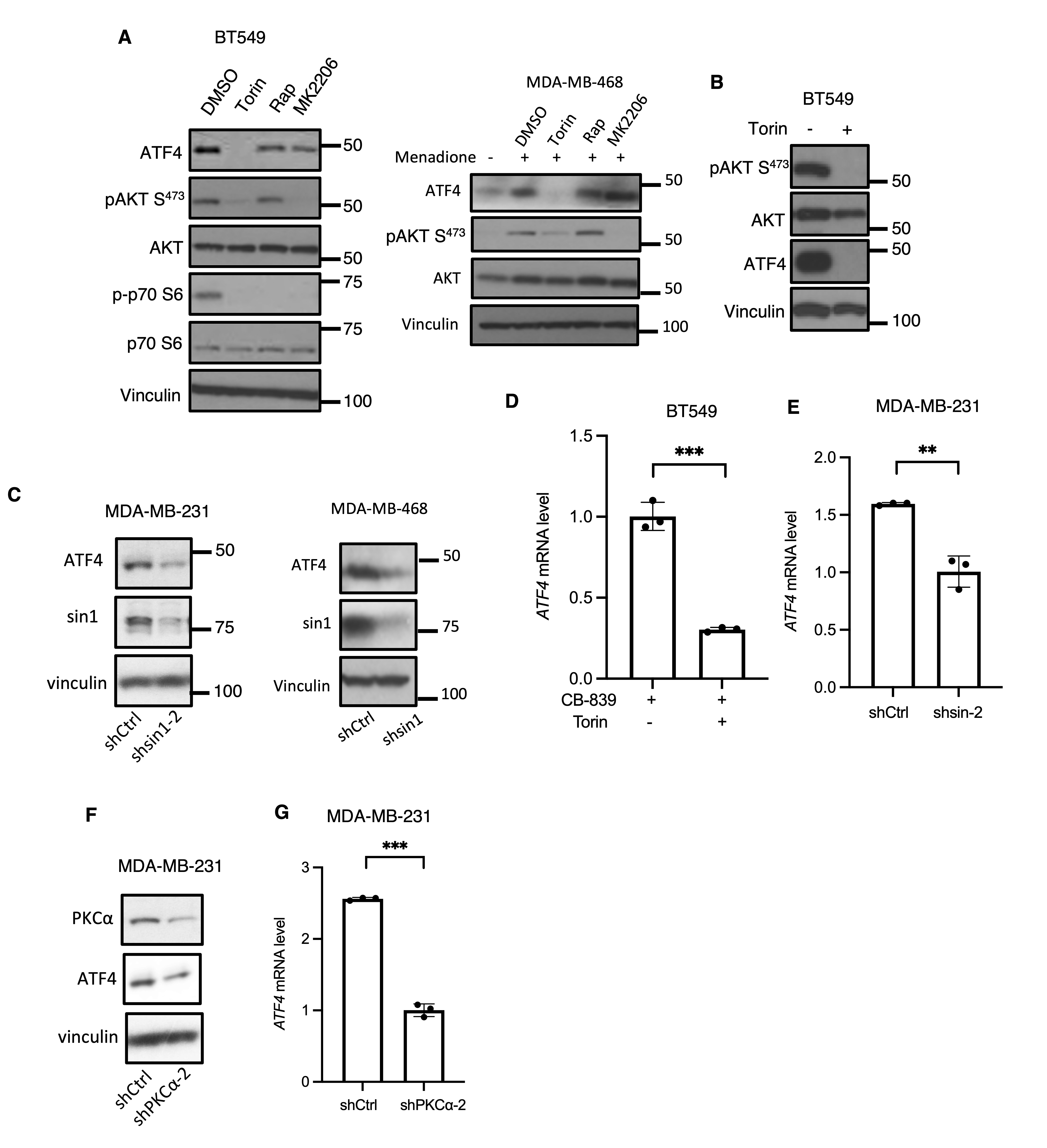


**Fig. S2. mTORC2 upregulates ATF4 transcription independently of AKT.**

**A** Western blot analysis of whole cell lysates (WCL) from BT549 and MDA-MB-468 cells treated with menadione (10 μM) for 2h with Torin1 (500 nM), Rapamycin (50 nM) or MK2206 (5 μM). Blots are representative of two independent experiments.

**B** Western blot analysis of WCL from BT549 cells treated with CB 839 (1 μM) ± Torin1 (500 nM) for 4h. Blots are representative of three independent experiments.

**C** Western blot analysis of WCL from control or *sin1* KD MDA-MB-231 and MDA-MB-468 cells cultured in glutamine-free medium (MDA-MB-231) or treated with CB 838 (MDA-MB-468) for 24 h. Blots are representative of two independent experiments.

**D** RT-qPCR quantification of *ATF4* mRNA in BT549 cells treated with CB 839 (1 μM) ± Torin1 (500 nM) for 24 h. Data shown are representative of three independent experiments and expressed as means ± SD for triplicate measurements.

**E** RT-qPCR quantification of *ATF4* mRNA in control or *sin1* KD MDA-MB-231 cells cultured in glutamine-free medium for 24 h.

**F** Western blot analysis of WCL of control and *PKCα* KD MDA-MB-231 cells treated with CB-839 (1 μM) for 24 h. Blots are representative of three independent experiments.

**G** RT-qPCR quantification of *ATF4* mRNA in control and *PKCα* KD MDA-MB-231 cells deprived of glutamine for 24 h.

*p<0.05, **p<0.01 and ***p<0.001 by Student’s t-test.


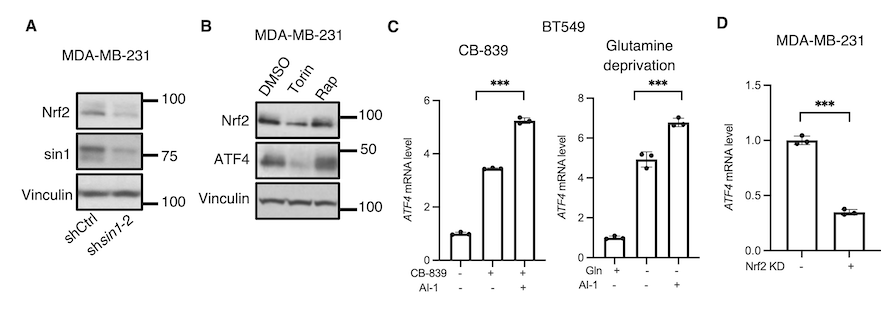


**Fig. S3. mTORC2-PKC-Nrf2 axis promotes ATF4**

**A** Western blot analysis of whole cell lysates (WCL) from control or *sin1* KD MDA-MB-231 cells treated CB 839 (1 μM) for 24 h. Blots are representative of two independent experiments.

**B** Western blot analysis of WCL from MDA-MB-231 cells treated CB 839 (1 μM) ± Torin1 (500 nM) or Rapamycin (50 nM) for 24 h. Blots are representative of two independent experiments.

**C** RT-qPCR quantification of *ATF4* mRNA in BT549 cells treated with CB 839 (1 μM) or deprived of glutamine ± AI-1 (10 mM) for 24 h. Data shown are representative of two independent experiments and expressed as means ± SD for triplicate measurements.

**D** RT-qPCR quantification of *ATF4* mRNA in control and *NRF2* KD MDA-MB-231 cells treated with menadione (10 μM) for 8h. Data shown are representative of three independent experiments and expressed as means ± SD for triplicate measurements. *p<0.05, **p<0.01 and ***p<0.001 by Student’s t-test.


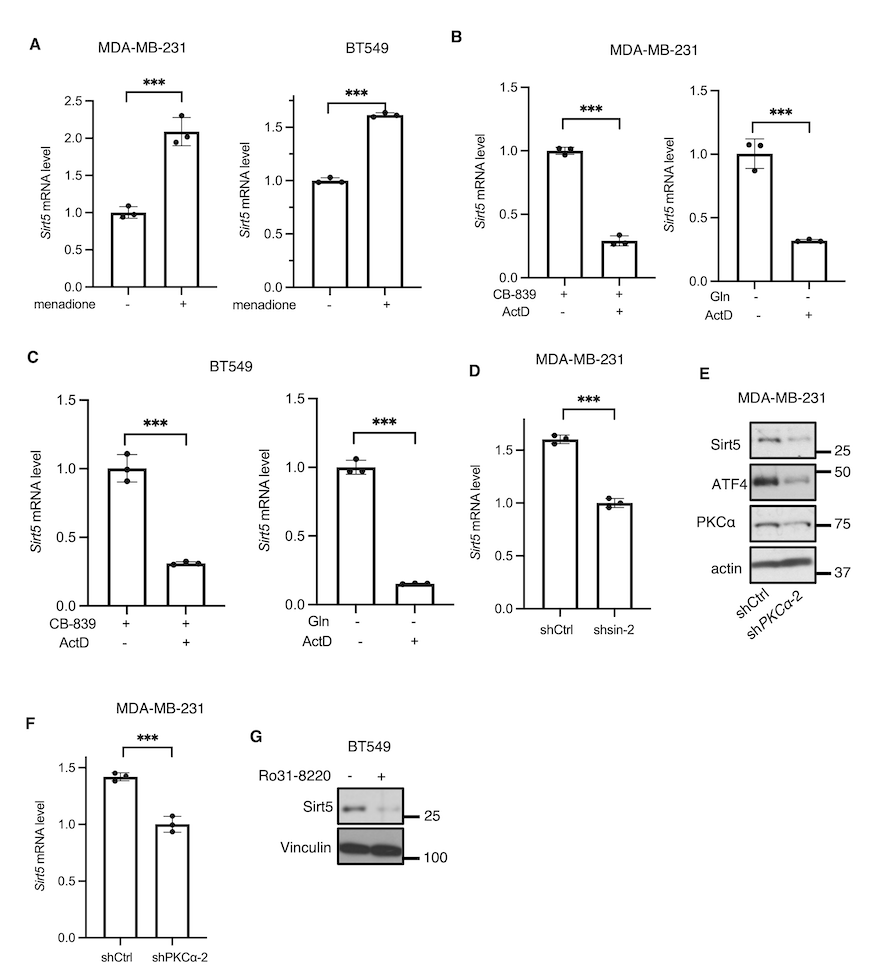


**Fig. S4. The regulation of Sirt5 expression parallels ATF4.**

**A** RT-qPCR quantification of *Sirt5* mRNA in MDA-MB-231 cells ± menadione (10 μM) and in BT549 cells ± menadione (2 μM) for 24 h. Data shown are representative of two independent experiments and expressed as means ± SD for triplicate measurements.

**B** RT-qPCR quantification of *Sirt5* mRNA in MDA-MB-231 cells treated with CB 839 (1 μM) or without glutamine ± ActD (1 μg/mL) for 24 h. Data shown are representative of two independent experiments and expressed as means ± SD for triplicate measurements.

**C** RT-qPCR quantification of *Sirt5* mRNA in BT549 cells treated with CB 839 (1 μM) or without glutamine ± ActD (1 μg/mL) for 24 h. Data shown are representative of two independent experiments and expressed as means ± SD for triplicate measurements.

**D** RT-qPCR quantification of *Sirt5* mRNA in control and *sin1* KD MDA-MB-231 cells treated without glutamine for 24 h.

**E** Western blot analysis of whole cell lysates (WCL) from control or *PKC α* KD MDA-MB-231 cells cultured in glutamine free condition for 24 h. Blots are representative of two independent experiments.

**F** RT-qPCR quantification of *Sirt5* mRNA in control and *PKCα* KD MDA-MB-231 cells treated without glutamine for 24 h.

**G** Western blot analysis of WCL from BT549 cells were treated with CB 839 (1 μM) ± Ro 31-8220 (5 μM) for 24 h. Blots are representative of two independent experiments.

**
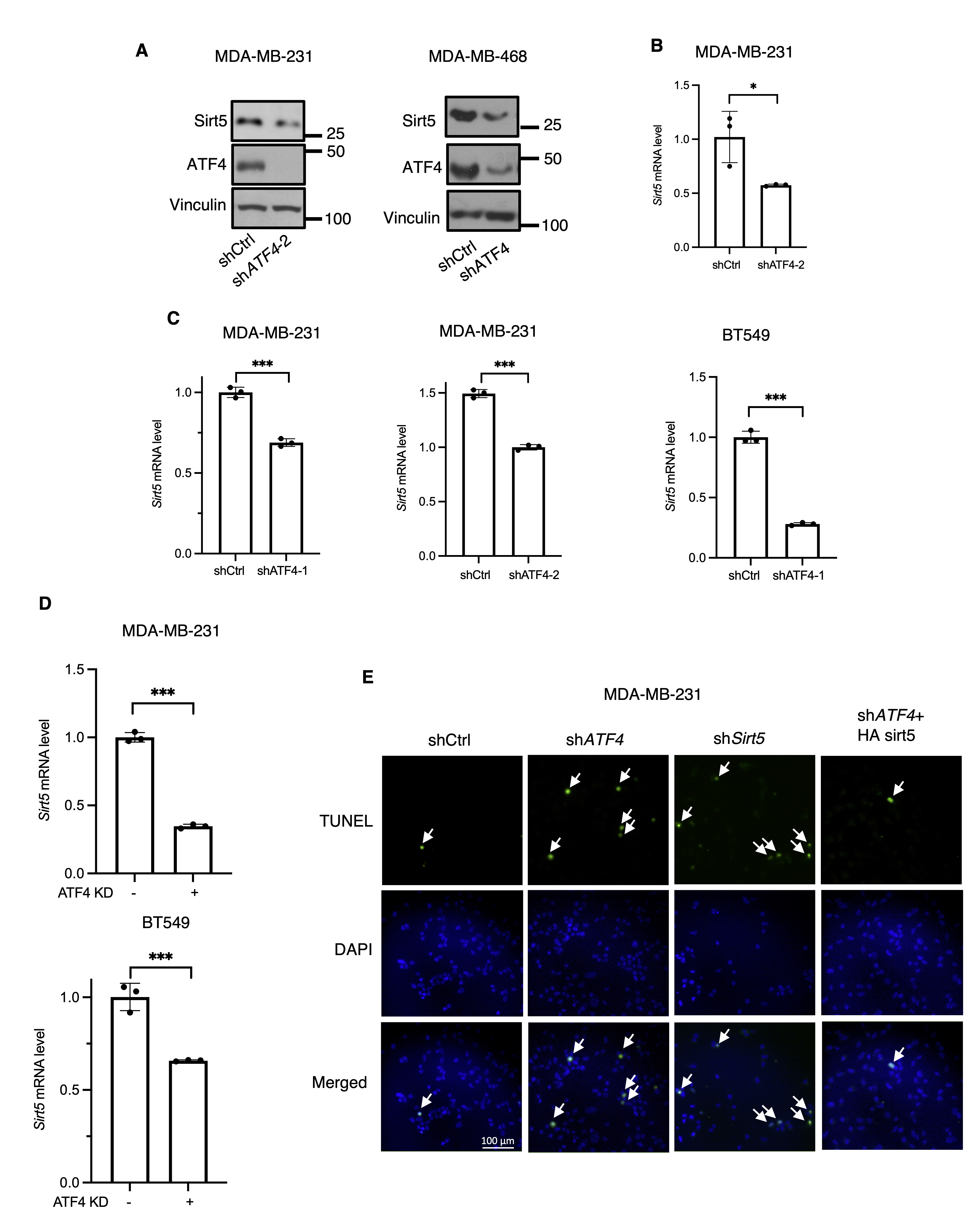
**

**Fig. S5. *Sirt5* is a transcriptional target of ATF4 necessary for survival.**

**A** Western blot analysis of whole cell lysates (WCL) from control or *ATF4* KD MDA-MB-231 and MDA-MB-468 cells treated with CB-839 (1 μM) for 24 h. Blots are representative of two independent experiments.

**B** RT-qPCR quantification of *Sirt5* mRNA in control and *ATF4* KD MDA-MB-231 cells treated with CB-839 (1 μM) or 24 h.

**C** RT-qPCR quantification of *Sirt5* mRNA in control and *ATF4* KD MDA-MB-231 and BT549 cells cultured in glutamine free media for 24 h. Data shown are representative of three (MDA-MB-231) or two (BT549) independent experiments and expressed as means ± SD for triplicate measurements.

**D** RT-qPCR quantification of *Sirt5* mRNA in control and *ATF4* KD MDA-MB-231 and BT549 cells treated with menadione for 24 h. Data shown are representative of two independent experiments and expressed as means ± SD for triplicate measurements.

**E** TUNEL assay showing that *ATF4* or *Sirt5* knockdown-induced cell death. MDA-MB-231 cells were cultured in glutamine and serum free media for 48h. Cell death was measured by TUNEL (green fluorescence). Nuclear location was determined by DAPI staining (blue fluorescence). Data shown are representative of two independent experiments.

**
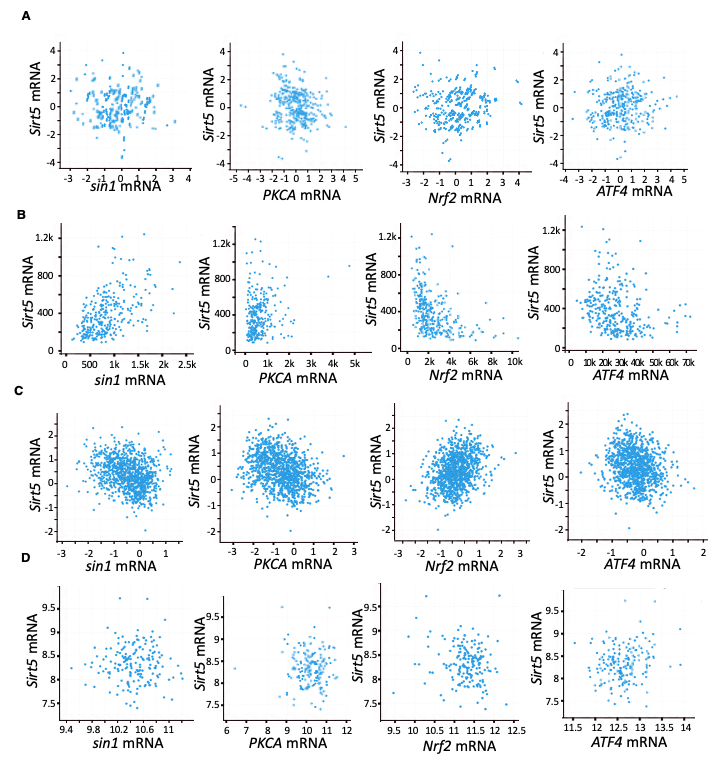
**

**Fig S6. TCGA analysis of pathway genes in lung, lymphoid, ovarian/fallopian tube, and pancreatic cancers.**

**A** Scatter plots show Sirt5 mRNA levels do not correlate with *sin1* (Spearman: 0.12 Pearson: 0.05), *PKCA* (Spearman: -0.17 Pearson: -0.15), *NRF2* (Spearman: 0.14 Pearson: 0.12) and *ATF4* (Spearman: 0.12 Pearson: 0.10) mRNA levels in lung cancer.

**B** Scatter plots show Sirt5 mRNA levels do not correlate with *sin1* (Spearman: 0.52 Pearson: 0.52), *PKCA* (Spearman: 0.19 Pearson: 0.19), *NRF2* (Spearman: -0.53 Pearson: -0.43) and *ATF4* (Spearman: -0.32 Pearson: -0.24) mRNA levels in lymphoid cancer.

**C** Scatter plots show Sirt5 mRNA levels do not correlate with *sin1* (Spearman: -0.29 Pearson: -0.27), *PKCA* (Spearman: -0.33 Pearson: -0.31), *NRF2* (Spearman: 0.30 Pearson: 0.30) and *ATF4* (Spearman: -0.21 Pearson: -0.20) mRNA levels in ovarian and fallopian tube cancer.

**D** Scatter plots show Sirt5 mRNA levels do not correlate with *sin1* (Spearman: 0.00 Pearson: 0.00), *PKCA* (Spearman: -0.02 Pearson: -0.02), *NRF2* (Spearman: -0.12 Pearson: 0.14) and *ATF4* (Spearman: 0.16 Pearson: 0.17) mRNA levels in pancreatic cancer.
