# Supplementary material for: Elucidation of an mTORC2-PKC-NRF2 pathway that sustains the ATF4 stress response and identification of Sirt5 as a key ATF4 effector
Source: Cell Death Discov. 2022 Aug 13;8:357. doi: 10.1038/s41420-022-01156-5 (PMC9376072; doi:10.1038/s41420-022-01156-5)
Supplement: Supplementary file 2 — Uncropped Western Blot [file 41420_2022_1156_MOESM2_ESM.docx]

**
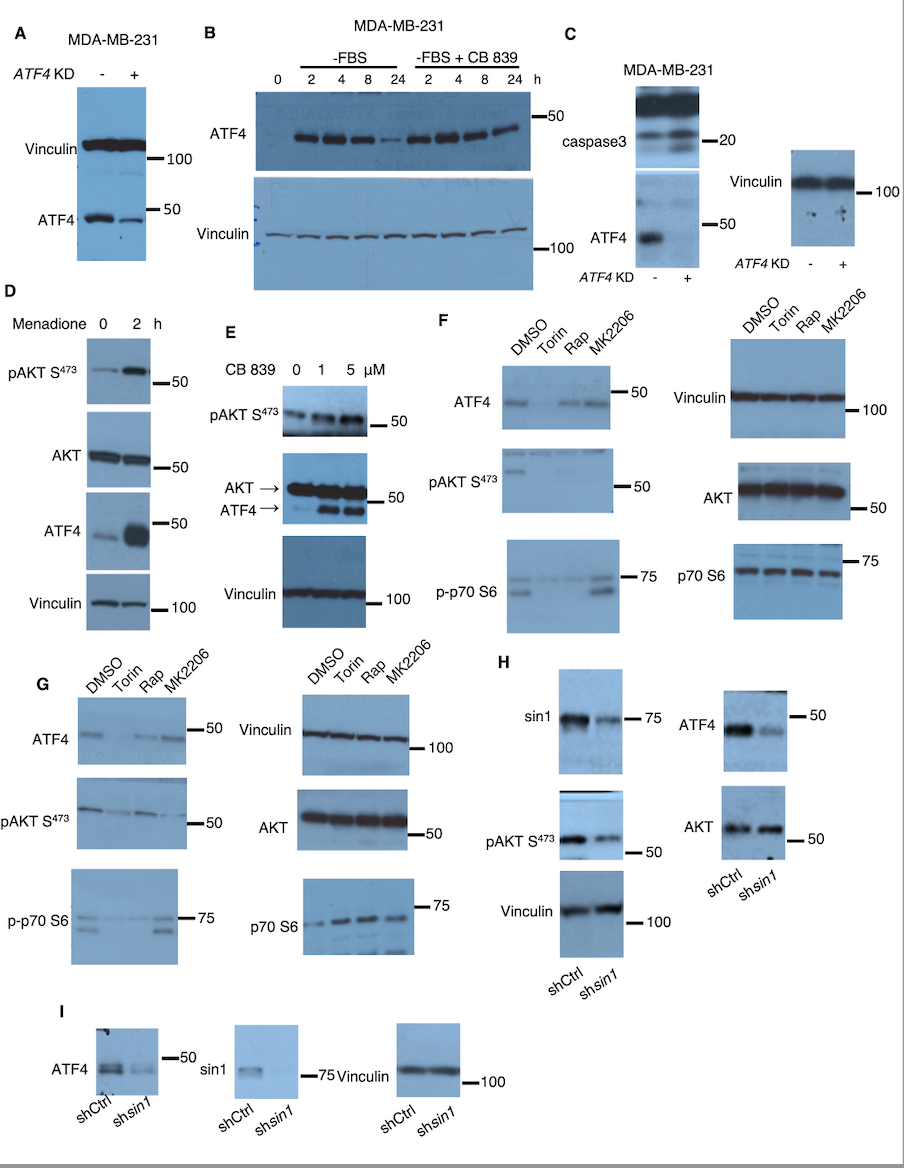
**

**Supplementary Figure Uncropped western blot scans.**

**A** WCL from control or *ATF4* KD MDA-MB-231 cells.

**B** MDA-MB-231 cells ± CB-839 (1 μM) at indicated times.

**C** MDA-MB-231 cells cultured in glutamine free condition for 48 h.

**D** MDA-MB-231 cells treated with menadione (50 μM) for 2 h.
**E** MDA-MB-231 cells treated with CB-839 for 24 h.

**F, G** MDA-MB-231 cells treated with CB-839 (1 μM) (F) or without glutamine (G) ± Torin1 (500 nM), Rapamycin (50 nM) or MK2206 (5 μM) for 24 h.

**H, I** WCL from control or *sin1* KD MDA-MB-231 cells cultured in glutamine-free medium (H) or treated with CB-839 (1 μM) (I) for 24 h.


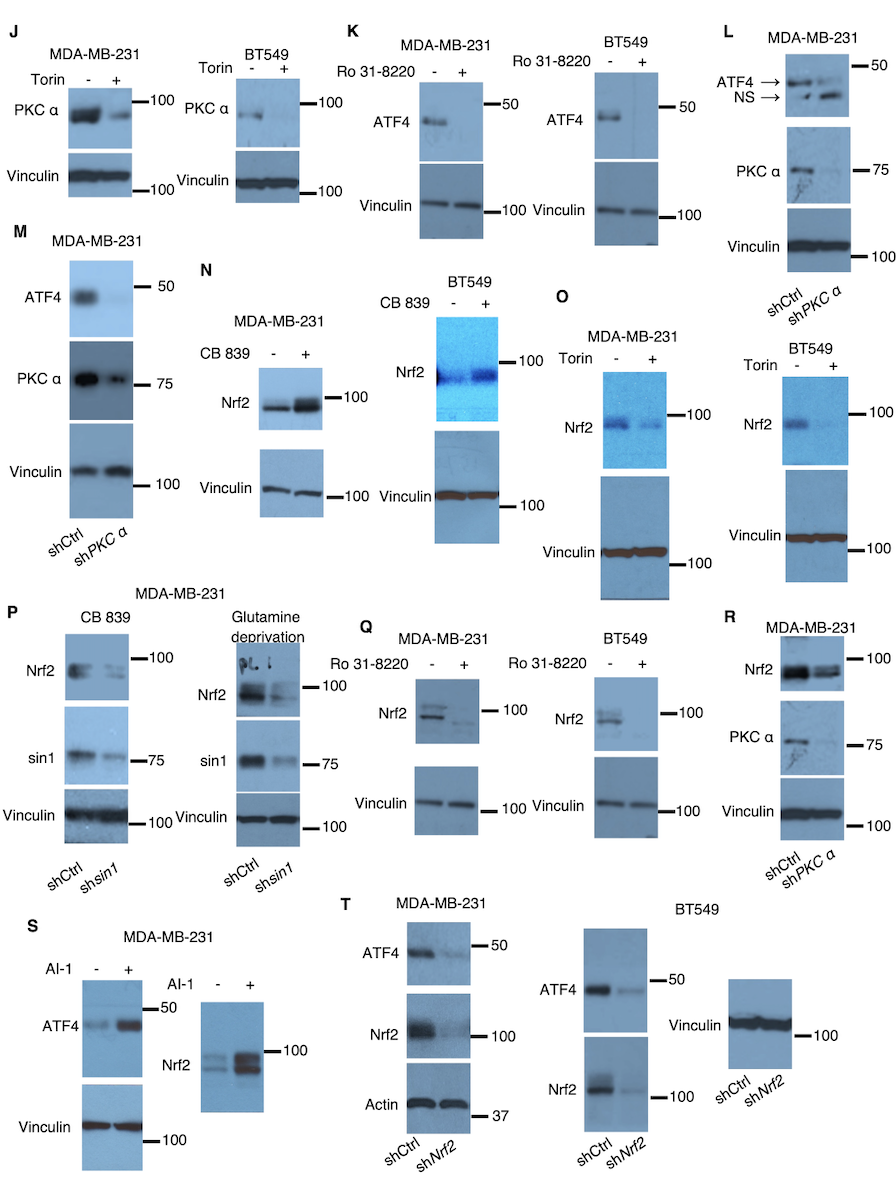


**Supplementary Figure Uncropped western blot scans (continued).**

**J** MDA-MB-231 and BT549 cells treated with CB-839 (1 μM) ± Torin1 (500 nM) for 24 h.

**K** MDA-MB-231 and BT549 cells treated with CB-839 (1 μM) ± Ro31-8220 (5 μM) for 24 h.
**L** WCL from control and *PKCα* KD MDA- MB-231 cells treated with CB-839 (1 μM) for 24 h.

**M** WCL from control and *PKCα* KD MDA- MB-231 cells deprived of glutamine for 24 h.

**N** MDA-MB-231 and BT549 cells ± CB-839 (1 μM) for 24 h.
**O** MDA-MB-231 and BT549 cells treated with CB-839 (1 μM) ± Torin1 (500 nM) for 24 h.

**P** WCL from control or *sin1* KD MDA-MB-231 cells treated with CB-839 (1 μM) or deprived of glutamine for 24 h.

**Q** MDA-MB-231 and BT549 cells treated with CB-839 (1 μM) ± Ro31-8220 (5 μM) for 24 h.

**R** WCL from control or *PKCα* KD MDA-MB-231 cells treated with CB-839 (1 μM) for 24 h.
**S** MDA-MB-231 cells treated with CB-839 (1 μM) ± AI-1 (10 mM) for 12 h.

**T** WCL from control and *Nrf2* KD MDA-MB-231 and BT549 cells treated with CB-839 (1 μM) for 24 h.


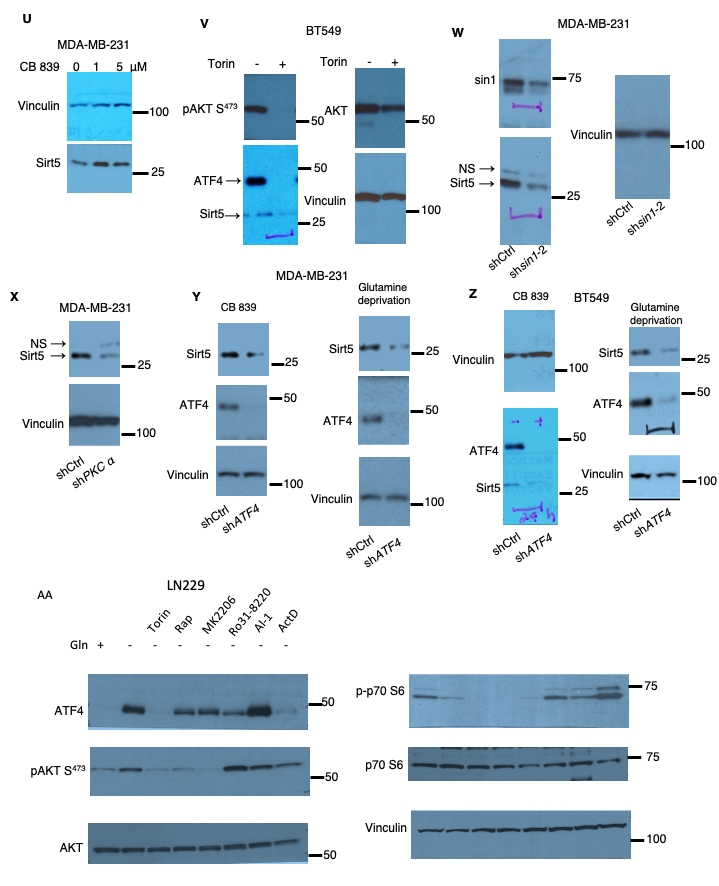


**Supplementary Figure Uncropped western blot scans (continued).**

**U** MDA-MB-231 cells ± CB-839 for 24 h.

**V** BT549 cells treated with CB-839 (1 μM) ± Torin1 (500 nM) for 4 h.

**W** WCL from control or *sin1* KD MDA-MB-231 cells treated with CB-839 (1 μM) for 24 h.

**X** WCL from control and *PKCα* KD MDA- MB-231 cells treated with CB-839 (1 μM) for 24 h.

**Y** WCL from control or *ATF4* KD MDA-MB-231 cells treated with CB-839 (1 μM) for 8 h or without glutamine for 24 h.

**Z** WCL from control or *ATF4* KD BT549 cells treated with CB-839 (1 μM) or without glutamine for 24 h.

**AA** Western blot analysis of WCL from LN229 cells treated with Torin1 (500 nM), Rapamycin (50 nM), MK2206 (5 μM), Ro31-8220 (1 μM), AI-1 (10 mM) or ActD (1μg/mL) in serum/glutamine free medium for 24h.

**
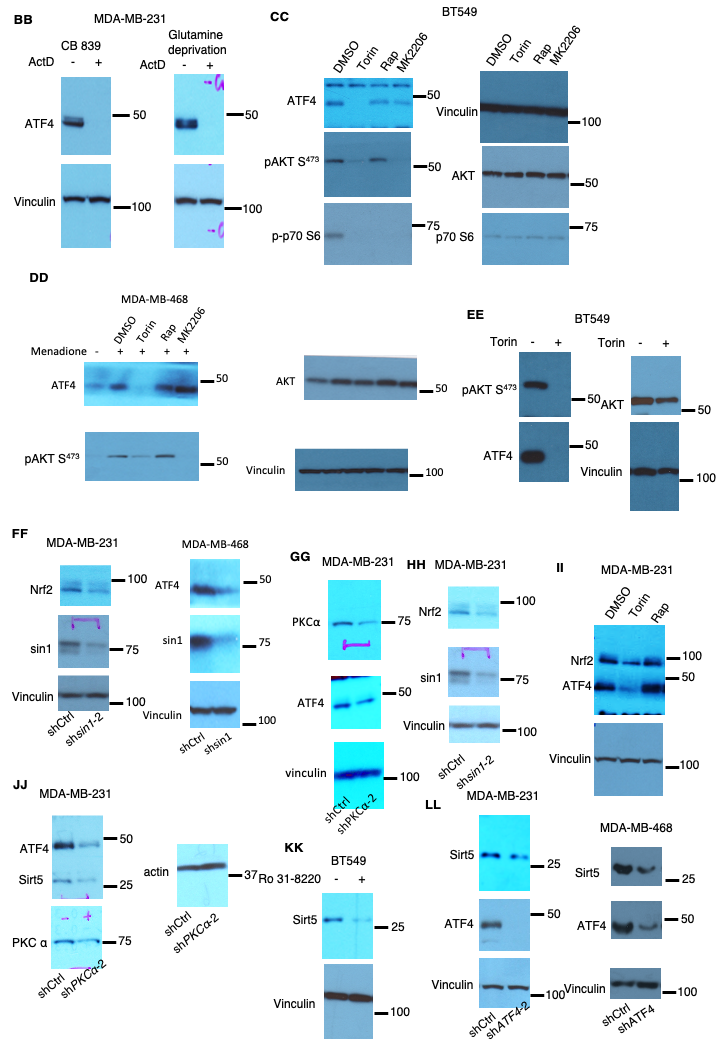
**

**Supplementary Figure Uncropped western blot scans (continued).**

**BB** MDA-MB-231 cells treated with CB-839 (1 μM) or without glutamine ± ActD (1 μg/mL) for 24 h.

**CC** MDA-MB-231 cells treated with menadione (10 μM) for 2h with Torin1 (500 nM), Rapamycin (50 nM) or MK2206 (5 μM).

**DD** WCL from BT549 and MDA-MB-468 cells treated with menadione (10 μM) for 2h with Torin1 (500 nM), Rapamycin (50 nM) or MK2206 (5 μM).

**EE** BT549 cells treated with CB-839 (1 μM) ± Torin1 (500 nM) for 4h.

**FF** WCL from control or *sin1* KD MDA-MB-231 and MDA-MB-468 cells cultured in glutamine-free medium (MDA-MB-231) or treated with CB 838 (MDA-MB-468) for 24 h.

**GG** WCL of control and *PKCα* KD MDA-MB-231 cells treated with CB-839 (1 μM) for 24 h.

**HH** WCL from control or *sin1* KD MDA-MB-231 cells treated CB 839 (1 μM) for 24 h.

**II** MDA-MB-231 cells treated CB-839 (1 μM) ± Torin1 (500 nM) or Rapamycin (50 nM) for 24 h.

**JJ** WCL from control or *PKCα* KD MDA-MB-231 cells cultured in glutamine free condition for 24 h.

**KK** BT549 cells were treated with CB-839 (1 μM) ± Ro31-8220 (5 μM) for 24 h.

**LL** WCL from control or *ATF4* KD MDA-MB-231 and MDA-MB-468 cells treated with CB-839 (1 μM) for 24 h.
